# Success criteria comparison of eight implemented projects to improve the planning, design, and construction of floodplain wetlands

**DOI:** 10.1371/journal.pwat.0000426

**Published:** 2026-01-16

**Authors:** Robert J. Hawley, Chris Nietch, Donnie Knight, Shelby Acosta, Kurt Cooper, Nora Korth, Abi Raetz, John McManus, Rebecca McClatchy, Jake Hahn, Hannah Lubbers, Laura Lair, Ryan J. Winston

**Affiliations:** 1 Sustainable Streams, Louisville, Kentucky, United States of America; 2 Office of Research and Development, United States of America Environmental Protection Agency, Cincinnati, Ohio, United States of America; 3 Partners for Fish and Wildlife Program, United States of America Fish and Wildlife Service, Dayton, Ohio, United States of America; 4 Clermont Soil and Water Conservation District, Owensville, Ohio, United States of America; 5 Adams-Clermont Solid Waste District, Batavia, Ohio, United States of America; 6 Office of Environment & Sustainability, Cincinnati, Ohio, United States of America; 7 Department of Food, Agricultural, and Biological Engineering, Department of Civil, Environmental, and Geodetic Engineering, Ohio State University, Columbus, Ohio, United States of America

## Abstract

Prior to mass deforestation of uplands and drainage efforts in lowlands, floodplain wetlands were abundant in river valleys. Deforestation of steep slopes and the associated accumulation of alluvial sediment in valley bottoms have left floodplains much drier and largely disconnected from their adjacent rivers, restricting the ecosystem and societal benefits floodplains can provide. By exporting alluvial sediments from floodplains, long buried wetlands can be restored with improved connectivity to stream networks. Such floodplain wetland restoration efforts can provide benefits at orders-of-magnitude lower costs than conventional stormwater control measures (SCMs) by minimizing pipes and other hardened infrastructure. Particularly in urban and suburban watersheds, floodplains are often one of the last open areas to locate SCMs that have the potential to intercept large volumes of runoff. They can also be coupled with recreational opportunities or urban canopy restoration programs, expanding the societal benefits of and opportunities to fund floodplain wetland restoration. This paper presents insights from planning, design, modeling, and construction of eight floodplain wetland restoration projects. The eight projects were compared using success criteria that spanned two themes: 1) maximizing the benefits that the floodplain wetland can provide and 2) maximizing the economic efficiency and durability of the project. As a part of the comparison, we note characteristics for identifying good sites for floodplain wetlands, design optimization strategies for various project goals (e.g., nutrient reductions vs. offloading excessively erosive streamflow vs. flood reduction), and construction sequencing approaches for efficiency and cost minimization. Removing alluvium from floodplains can provide expanded flood storage and lowered flood elevations, restored off-channel habitat for fish/birds, improved water quality, reduced erosion/biotic disturbance for mussels/macroinvertebrates, and, in some cases, the economic benefits of an abundant source of high-quality topsoil for farmers, landscapers, and developers.

## Introduction

Agrarian societies have been manipulating floodplains for millennia [1–3]. Arguably one of humanity’s most enduring floodplain impacts was associated with periods of widespread hillslope deforestation that coincided with increased delivery of water and sediment to alluvial valleys leading to sedimentation and increased flooding [4,5]. With the import of the sweet potato and maize, and the associated population expansion that further increased food demand, the cultivated area in China was estimated to have approximately tripled between 1700 and 1850 [6,7]. As crop cultivation replaced forests on steep slopes, the decreased canopy interception, evapotranspiration, and root strength increased the runoff of both water and sediment [7,8]. The associated flooding and sedimentation in the lowlands decreased crop yields in historically productive valleys, leading to further expansion of cultivation into even more erosion-prone areas [8] and extensive dike networks, channelization and floodplain drainage in river valleys like the Huang He [9].

The pattern of deforestation, erosion, flooding, sedimentation, and crop destruction reportedly occurred in at least one large society in North American circa 950–1250 at Cahokia [10]; however, historic American societies were more commonly associated with sustainable assemblages of cultivated areas balanced with buffering forests that were managed for game, nut and fruit production [11].

By contrast, the steel tools of European societies facilitated the pattern of deforestation, erosion, sedimentation, and flooding in essentially every land touched by European colonization [4], with ~10-fold increases in landscape erosion rates in North America after Europeans expanded into the continent [12]. Indeed, Europeans were so accustomed to their highly manipulated floodplains that early settlers were surprised by how different the North American streams and landscapes were from their English equivalents, with “a seemingly endless patchwork of bogs, marshes, grassy ponds, seasonally flooded meadows, and slow-moving streams” [4]. Beavers transformed rushing streams “into a series of broad pools and mucky wetlands linked by shallow, multiply branched channels... [Indigenous peoples] regarded this as a fine thing—easier to take a canoe through a set of pools than a narrow, quick-flowing stream” [4]. Over the next several centuries, European-influenced societies collectively transformed North American floodplains, reducing their ability to provide flood control, assimilate nutrients, and provide productive aquatic habitats for fish and other wildlife, and reducing subsidies to terrestrial animals [13]. Drainage and development of floodplains and wetlands [14], construction of levees [15], channelization [16], extirpation of beaver [17], and the accumulation of large amounts of post-settlement alluvium [5] have all resulted in less available space for flood waters while at the same time concentrating the energy of the flows into a smaller area within the main channel, exacerbating erosion [18] and flood elevations [19]. Today, natural floodplain areas continue to be converted into developed and agricultural lands, with an estimated 600,000 km^2^ of floodplain losses globally from 1992 to 2019 [20]. Superimposed on this physically degraded floodplain setting is the recently supercharged atmosphere capable of holding more moisture and delivering larger and more intense precipitation events [21].

Renovating floodplains by removing alluvium has the potential to revitalize buried floodplain wetlands, restoring the associated ecosystem benefits [22] and making our waterways more climate resilient [23]. For the purposes of this paper we broadly define floodplain wetlands as any off-channel aquatic habitat (e.g., oxbow, billabong, wetland, slough, depression, etc.) in a stream or river floodplain with the potential to support aquatic, wetland, and floodplain functions such as sustaining emergent macrophytes and flood tolerant woody trees and shrubs, providing seasonal or permanent habitat for amphibians, fish, or aquatic birds, and assimilating nutrients. In this light, the term does not necessarily fit requirements of a jurisdictional wetland habitat with fully developed wetland soils [24].

This paper is structured into two parts. First, we draw from literature and experience to develop a set of success criteria to evaluate the benefits, economic efficiency and durability of constructed floodplain wetlands. Second, we use these success criteria to compare eight floodplain wetland projects that have been implemented by the authors. Some of the potential benefits of floodplain wetlands include flood reduction, habitat expansion, nutrient assimilation, erosion reduction, and baseflow augmentation [25]. Some of the planning, design, and construction insights that increase economic efficiency and project durability include site selection and construction sequencing that minimize extraneous costs (e.g., minimizing soil haul off, establishing herbaceous cover in the excavated wetland prior to constructing the hydraulic connection to the river, etc.) and ensuring stable connections between the adjacent river and wetland. Our synthesis incorporates data, modeling, costs, and experiential insights with a goal of facilitating greater situational awareness for others as they tailor their own projects. It is our hope that these lessons learned from constructed projects can better enable planners, administrators, reviewers, designers, and contractors to implement cost-effective, resilient, and sustainable floodplain wetland projects with numerous co-benefits to natural resources and society.

## General project descriptions and methods

This synthesis draws upon literature insights and experience from eight constructed floodplain wetland projects that were installed between 2015 and 2023 along streams and rivers in the greater region of southwest Ohio and Northern Kentucky (Table 1). The region has a humid continental climate [26] with annual rainfall of ca. 1,000 mm. A kmz file of the eight wetland locations is available as S1 Data in S1 File in the supplementary materials.

A combination of standard hydrology and hydraulic modeling was used to inform the design and permitting of the eight projects, including US Geological Survey (USGS) gauge data where available, USGS Streamstats, the HydroCAD software, 1-dimensional hydraulic modeling in the US Army Corps of Engineers (USACE) Hydraulic Engineering Center River Analysis System (HEC-RAS) software, and 2-dimentional hydraulic modeling in the program RiverFlow2D. In some cases, highly calibrated HEC-RAS models created by a regional flood control district were available. Designs were optimized for primary, secondary, and ancillary project objectives (Table 1) based on stakeholder input, funding source criteria, site constraints, permitting requirements, and other factors.

For our eight projects, permitted engineering designs included standard grading plans with proposed and existing contours, river-wetland connections such as channels or rock weirs, bioengineering details, and planting plans developed in the AutoCAD software. Connection armoring, if necessary, was designed using standard rock sizing methods relevant for the application (e.g., rock chutes [27], etc.). Large wood anchoring via burial was designed via the Colorado State University Large Wood Tool [28]. In some cases, the bulk of the wetland excavation could be completed by a conceptual-level grading plan based on publicly available LiDAR contours as opposed to more precise terrestrial surveys of the site. In all cases, required permitting correspondence (e.g., flood control, water quality, endangered species, historic preservation, etc.) was completed prior to placing any equipment or fill within a jurisdictional waterway, and standard construction erosion control practices were followed (e.g., gravel construction entrances to clean construction equipment tires prior to entering public streets, providing temporary cover on disturbed soils, etc.).

**Fig 1.**
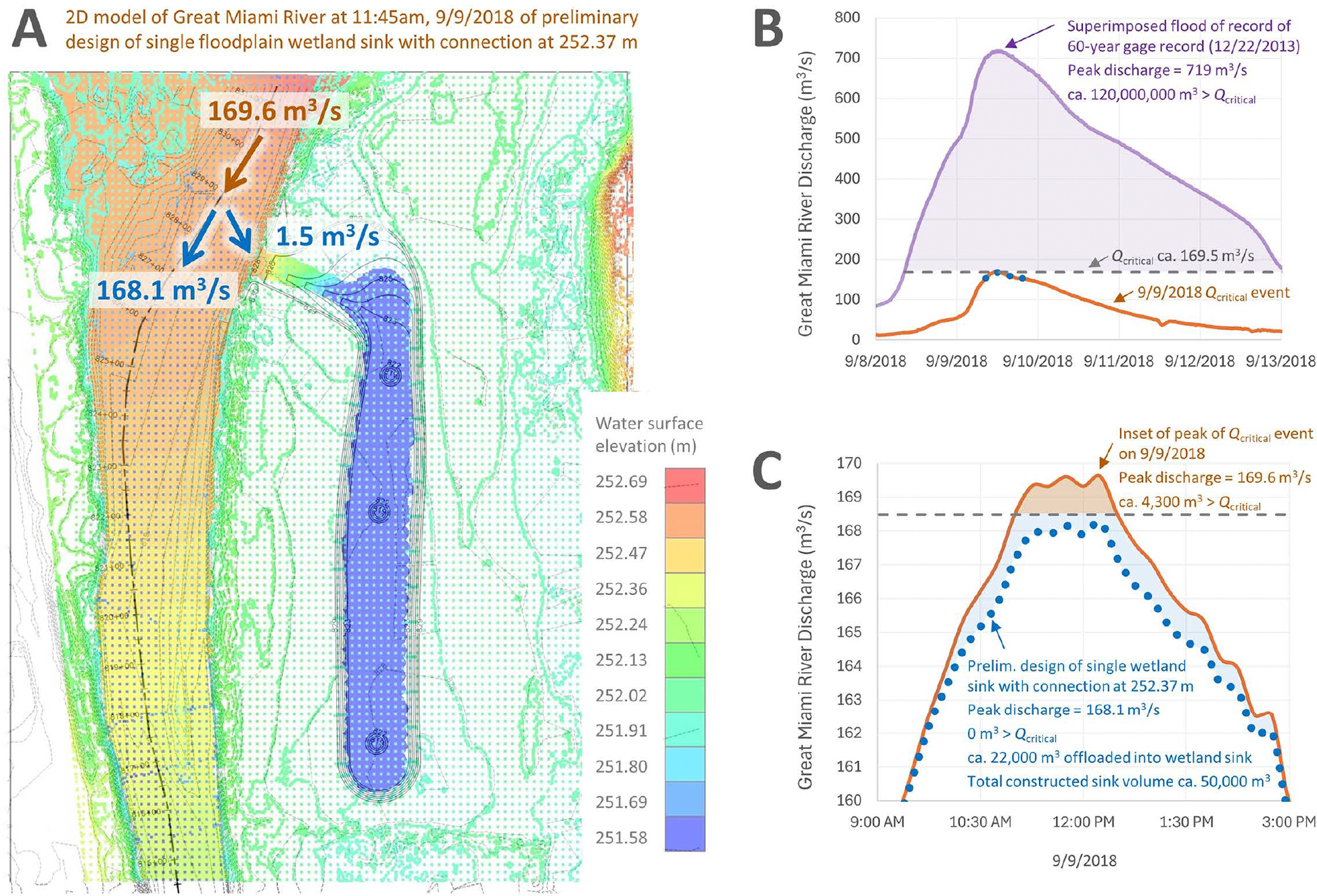
Two-dimensional modeling of a preliminary design of a single floodplain wetland sink at Duke Park (A) underscores the potential erosion reduction benefits for events that barely exceed the critical discharge for streambed erosion (*Q*_critical_). The total constructed volume of multiple sinks of ca. 50,000 m^3^ at Duke Park would be quickly overwhelmed by largest recorded flow during the gauge record for the 60-yr inventory at USGS gauge #03262700 on the Great Miami River in Ohio (B), with a peak of 719 m^3^/s on 12/22/2013 and ca. 120,000,000 m^3^ of volume that exceeds *Q*_critical_. However, for bed-mobilizing events such as occurred 9/9/2018 that barely exceeded *Q*_critical_, the connection elevation and geometry can be optimized to offload flows that would otherwise contribute to streambed erosion (C), thereby extending the period of seasonal stability for freshwater benthic organisms that are vulnerable to streambed disturbance such as macroinvertebrates and early juvenile mussels. https://doi.org/10.1371/journal.pwat.0000426.g001

**Fig 2.**
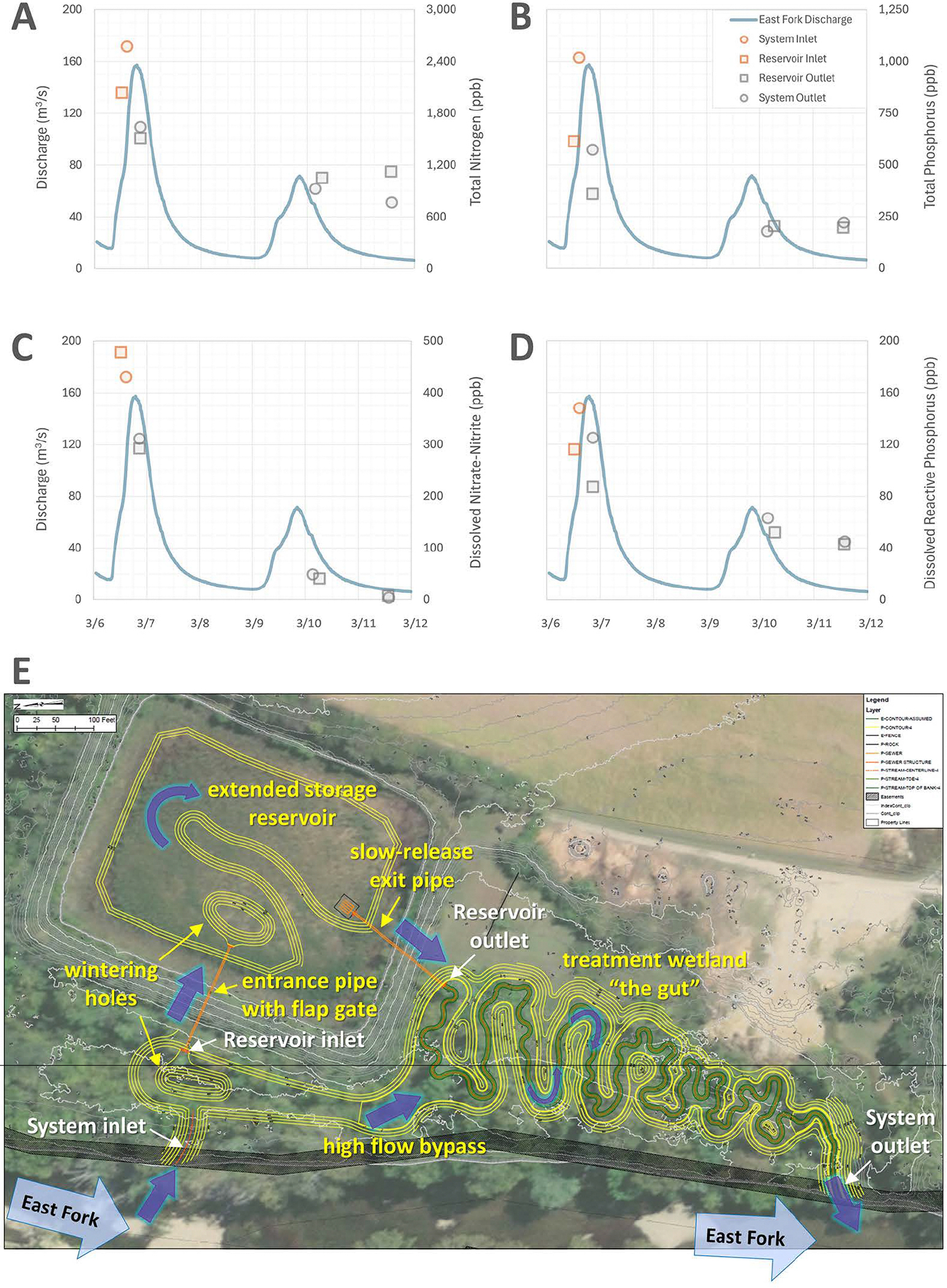
Nutrient species concentrations: Total Nitrogen (A), Total Phosphorus (B), Dissolved Nitrate-Nitrite (C) and Dissolved Reactive Phosphorus (D) sampled at treatment system monitoring points relative to the discharge hydrograph from a March 6, 2024-event at the Williamsburg floodplain wetland along the East Fork of the Little Miami River. White text on map (E) indicates sample locations, with wetland design features in yellow text and flow paths depicted by purple arrows. https://doi.org/10.1371/journal.pwat.0000426.g002

**Fig 3.**
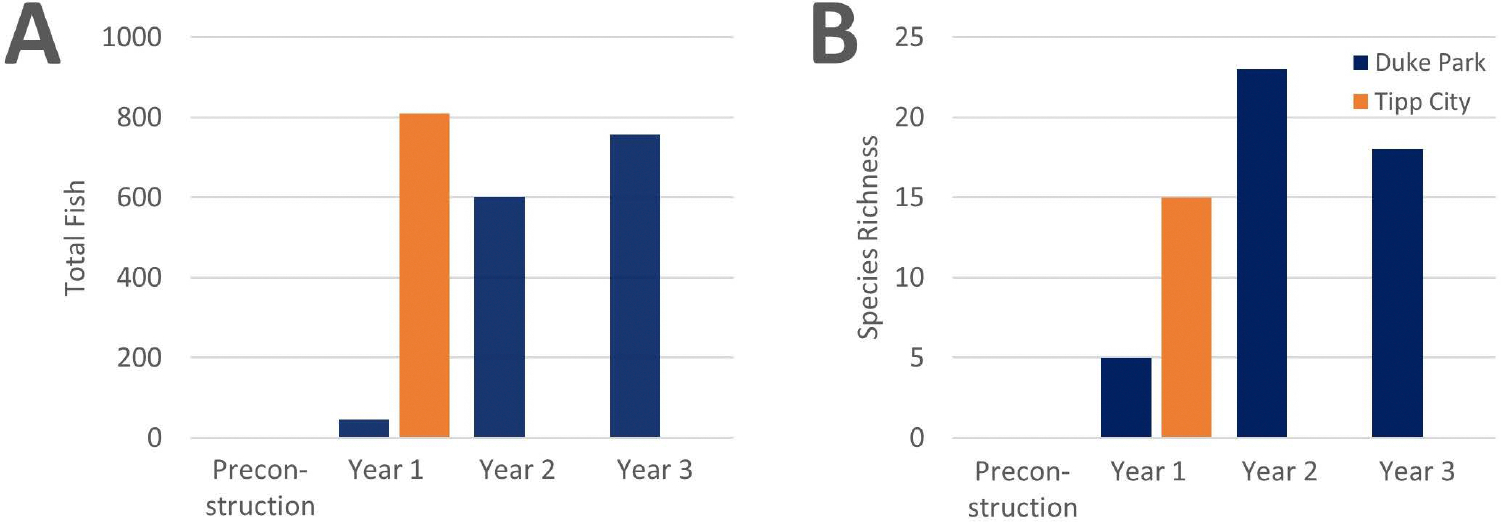
**Annual fish data prior to and after construction of Duke Park and Tipp City floodplain wetlands** for total fish (A) and species richness (B). Both sink-style wetlands are along the Great Miami River north of Dayton, OH. Duke Park was constructed in 2020, with Years 1 through 3 sampling occurring in 2021, 2022, and 2023, respectively. Tipp City was constructed in 2022, with the Year 1 sample occurring in 2023. https://doi.org/10.1371/journal.pwat.0000426.g003

Where available, construction costs are based on construction bids for our eight projects (Table 1). Duke Park, Rainbow Run, and Tipp City were constructed in phases using multiple funding sources, with some work completed by in-house staff and equipment rentals. Construction costs for those projects are therefore based on total grant expenditures/funding for the respective projects as opposed to external payments to construction contractors. At one of the wetlands, funding was available for event-based water quality data collected via autosamplers at the system inlet and outlet along with three other strategic locations throughout the system. Water quality samples were analyzed by project partners following standard methods and quality control procedures such as laboratory duplicates, matrix spikes, and other quality assurance measures. Fish samples were collected at two of the wetlands by experienced biologists following standard identification keys and equal length surveys (complete shoreline) to facilitate community survey comparisons across sites and sample years [29]. Plant inventories also referenced standard identification keys [30,31]. More details of methods are provided in the project reporting, supplementary materials, and associated publications referenced herein. Data for Fig 1, Fig 2, Fig 3 are available in S2 Data in S2 File.

## Success criteria - floodplain wetland benefits

In this section, floodplain wetland benefits are compiled from the literature and experience. The floodplain wetland benefits are organized by topics of flood attenuation, reduced stream erosion, prolonged baseflows, improved water quality, and habitat expansion. Some examples of benefits that are not discussed in detail include carbon sequestration [32] in soils and woody vegetation, educational opportunities, and the expansion or enhancement of greenspace areas.

### Flood attenuation

Many human societies have undergone periods of large-scale hillslope deforestation that coincided with the accumulation of large amounts of alluvial sediments in the floodplains [4]., including ca. 1–3 m on the sites of the eight projects. To put this into perspective, in the ~ 14,000 km^2^ watershed of the Great Miami River in southwest Ohio, the 100-yr floodplain is nearly 1,000 km^2^. If the entire present day floodplain area was 1.5 m lower, it would correspond to ~1.3 billion m^3^ of additional volume within the floodplain, which is comparable to the collective volume of ~1.2 billion m^3^ provided by the five major flood control reservoirs in the watershed [23]. Acknowledging that inline reservoirs attenuate flood peaks differently from well-connected floodplains, and that network-scale models would be needed to quantify these differences, their comparable volumes underscore the scale of the flood protection that may have been provided by floodplain wetlands under the pre-European settlement conditions in North America.

Floodplain wetlands can also serve as hydrologic ‘sinks’ to capture and store floodwaters by providing open storage volume below the river connection elevation. Given their alluvial floodplain setting, the water level in the wetland is likely to track with the groundwater level in the floodplain, which would vary seasonally and generally coincide with the river’s baseflow level [33]. During floods, the water level in most rivers rises much faster than the groundwater in the adjacent floodplain [34], such that floodwaters have the freedom to spill over into a lower elevation wetland once the river stage reaches the connection elevation (Fig 1A). In this sense, floodplain wetlands can offload a volume of water from the flood hydrograph that is comparable to the available storage volume within the wetlands. **Flood offloading volume** is therefore our first success criterion for evaluating the socio-economic and ecological benefits of restored floodplain wetlands.

During extreme events, the typical scale of the floodplain wetland volume will be quickly overwhelmed by the volume of water within the flood hydrograph (Fig 1B). However, by providing more available space to spread water out in a wetland area, water rises more slowly and flood elevations are lower [35]. This is a hydraulic benefit of floodplains. Furthermore, in relatively flat rivers with subcritical flow conditions, reduced water surface elevations in one location of the river will propagate reduced water surface elevations upstream [35], in contrast to levees that make water deeper upstream [19].

The distance of the propagated benefits upstream depends on numerous factors such as the configuration of the floodplain wetland as well as the river slope, bridge/levee constrictions, and other hydraulic influences. Thus, a hydraulic model is useful for evaluating and optimizing the design in each setting. In a flood risk reduction analysis for the City of Morehead, KY, the U.S. Army Corps of Engineers reported that a ca. 30 cm drop in the 100-yr water surface for a length of ca. 400 m could be achieved via a “high flow channel” flow-through design [36]. A high flow channel flow-through design includes a channel that is cut within an adjacent floodplain that conveys flows during relatively large events. By increasing the available area for hydraulic conveyance during high flow events, high-flow channels and similar flow-through designs can lower the water surface elevations relative to the pre-project conditions with less hydraulic conveyance area. By contrast, wetlands with a single upstream connection that are designed more exclusively as hydrologic sinks are likely to be less effective in reducing the elevation of the water surface during floods. Using standard flood permit modeling of the 100-yr discharge, the sink-style floodplain wetland on the Great Miami River at Duke Park was only modeled to drop the 100-yr water surface by 2 cm at the connection location [37] (S1 Report in S4 File). Compare this with projects that restore the entire valley bottom, coupling stream and floodplain wetland restoration via a multi-stage flow-through system that can also create appreciable drops in flood elevations. These hybrid-type wetland restorations combine different approaches to achieve multiple goals such as nutrient assimilation and flood attenuation. For instance, our East Fork Riparian project combined sinks/ depressions with valley-scale stream and floodplain restoration (Table 1) which resulted in an estimated average drop in the 100-yr water surface of 50 cm over the 335-m project reach, with a maximum drop near the upstream end of the project of nearly 100 cm [38] (S2 Report in S5 File). **Reduced flood elevation** is proposed as our second criterion for evaluating floodplain wetland projects.

### Reduced stream erosion

The hydrologic and hydraulic benefits for flood control can provide similar benefits in terms of reduced erosion in a river. The connection elevation between the floodplain wetland and the river can be optimized to offload flows that would scour a riverbed and erode banks even before flooding is a concern. Using an example hydrograph of an event that barely exceeded the critical discharge for streambed erosion (*Q*_critical_) [39], two-dimensional modeling or a simple spreadsheet model with a broad-crested weir equation [35] can be used to optimize the connection elevation, width, and slope to offload a portion of the hydrograph and eliminate exceedances of the *Q*_critical_ threshold (for an example, see Fig 1). Depending on the frequency and magnitude of *Q*_critical_ events in a system, a few optimized floodplain wetlands within a reach could substantially prolong the respite period between disturbance-inducing events. In a detailed analysis of Wabash River, which drains ca. 85,000 km^2^ in Ohio, Indiana, and Illinois, a ca. 10% reduction in the number of disturbance-inducing events could be achieved with just ca. 617,000 m^3^ of optimized floodplain wetland storage, with a conceptual-level construction cost of ca. $3M in 2021 dollars [40]. This could mean the difference for a benthic organism to complete a vulnerable portion of its life cycle when streambed entrainment can be a lethal disturbance [41,42]. **Reduced stream erosion** is our third criterion for evaluating floodplain wetland project success.

### Prolonged baseflows

Frequently inundated floodplain soils can retain water during flood events and slowly release water back to rivers when water levels in streams are lower than the adjacent water table [33,34]. Wetlands constructed as depressions can also store flows during offloading events and recharge groundwater after events recede [43]. The release of the stored water can occur naturally via infiltration processes or be managed by engineered outlet controls such as a series of perforated pipes protected by a gravel filter layer. A drainage system can be further optimized with a restrictor plate within the outlet pipe calibrated to release the stored volume over a desired time or below a maximum target discharge rate. The slow release induced by a 3-m long, 15-cm diameter, perforated underdrain protected by a 1-m tall by 1.3-m wide layer of gravel on the Shor Park stream daylighting and floodplain wetland project (Table 1) supplied enough baseflow to the receiving stream to convert it from a flashy ephemeral system with no fish into a system with perennially wet pools that were visually observed by the author to support fish during multiple site visits during summer low flow. These observations in expanded fish presence are consistent with fish observations reported downstream of a detention basin retrofit case study in Hebron, KY [44] where baseflow extensions were monitored by instrumentation [45]. **Prolonged baseflows** to the receiving stream network is our fourth success criterion for floodplain wetland restoration projects.

### Improved water quality

Intermediate and high flow events are associated with disproportionately high sediment and nutrient loads [46] that may be intercepted and retained by routing them into adjacent floodplain wetlands. The slow-flowing environments and longer retention times associated with flow paths through floodplain wetlands can induce sedimentation and promote nutrient assimilation [47,48]. Depending on the sediment composition, sand particles will likely settle out almost immediately upon entering the floodplain wetland, whereas lighter silts and clay particles require more time and may remain in suspension and be exported from the wetland unless additional controls are in place to extend detention times [49]. In the low-energy floodplain wetland at Williamsburg, ca. 0.3 meters of sediment were accumulated at the system inlet and sediment forebay/wintering hole (Fig 2E) during the first several offloading events (similar to the natural levee development process that is commonly observed along rivers) [50]. Sedimentation is also a mechanism for offloading adsorbed nutrients such as phosphorus [51].

Floodplain wetlands can also facilitate nutrient assimilation by providing flow paths and residence times that promote extended contact with vegetation and microbes that foster nutrient transformation, sequestration, or loss and which can be enhanced with carbon availability [47,48]. The denitrification process can be supported by providing a carbon source via the placement of woody material within constructed wetlands [52]. The carbon source combined with longer residence times has the potential to promote the anoxic-oxic interfaces that induce nitrate-nitrogen losses [53], as opposed to fast flowing environments where microbes can readily acquire dissolved oxygen from the water column. Denitrification from streams into the atmosphere reduces nutrient concentrations that cause eutrophication and toxic algal blooms [54].

The Williamsburg Wetland Project provides an example of a low energy flow-through system that was specifically optimized to facilitate nutrient assimilation. Data from a March 6, 2024-event underscore how concentrations of nitrogen and phosphorus are reduced as flow moves through the system (Fig 2). The system inlet is the upstream entry point to the wetland complex where flows begin to offload from the main river channel at an estimated discharge of 70 m^3^/s. The reservoir inlet is the point of inflow into the converted water supply reservoir that is used to store stormwater by detaining it with a flap gate and then slowly releasing water over a period of days through the reservoir outlet, where the slow release is monitored as flows discharge into “the gut”, a ~600-m meandering wetland channel with a ~0.025% slope. The system outlet monitors discharge from the gut back into river. During the high-flow portion of the hydrograph, nutrient concentration reductions of ca. 20–40% are observed between the inflow and outflow. However, after the high-flow portion of the event when the stored water within a built-in reservoir and in the wetland complex extends the detention time, nutrient concentration reductions range ca. 50–100% depending on the nutrient species (Fig 2). These data suggest that a slow-flowing wetland environment can facilitate significant levels of nutrient transformation and attenuation in addition to nutrient sequestration by sedimentation. **Improved water quality** is proposed as our fifth success criterion for evaluating floodplain wetland benefits.

### Habitat expansion

A wide range of habitats can be restored by reconnecting streams with floodplain wetlands. The construction of floodplain wetlands not only can restore emergent, scrub shrub, and forested wetland habitats [55], but they can also create deep pools to provide over-wintering habitats for a variety of fish species [56,57]. On two large floodplain wetland projects along the Great Miami River, fish abundance and diversity have increased in the overwintering pools (Fig 3). The Duke Park project was designed with a high connection elevation that was optimized for offloading erosive flows. The first year after construction, the permanent pool of the wetland only supported five fish species and less than 50 individuals. By the second year, the wetland pool supported over 20 species and more than 600 individuals, including several carnivorous game fish with populations that continued to expand in the third year (e.g., 1 and 47 largemouth bass (*Micropterus salmoides*), and 5 and 61 northern pike (*Esox lucius*) in years 2 and 3, respectively). However, a design allowing more frequent connectivity between the river and wetland facilitated by a lower connection elevation can speed up colonization. Although the lower connection elevation at the Tipp City Wetland was primarily designed to facilitate greater nutrient removal (Table 1), a secondary benefit of greater connectivity with the mainstem was achieved. Within just the first year after construction, the wetland had already been colonized by 15 species and over 800 individuals, including one largemouth bass.

**Fig 4.**
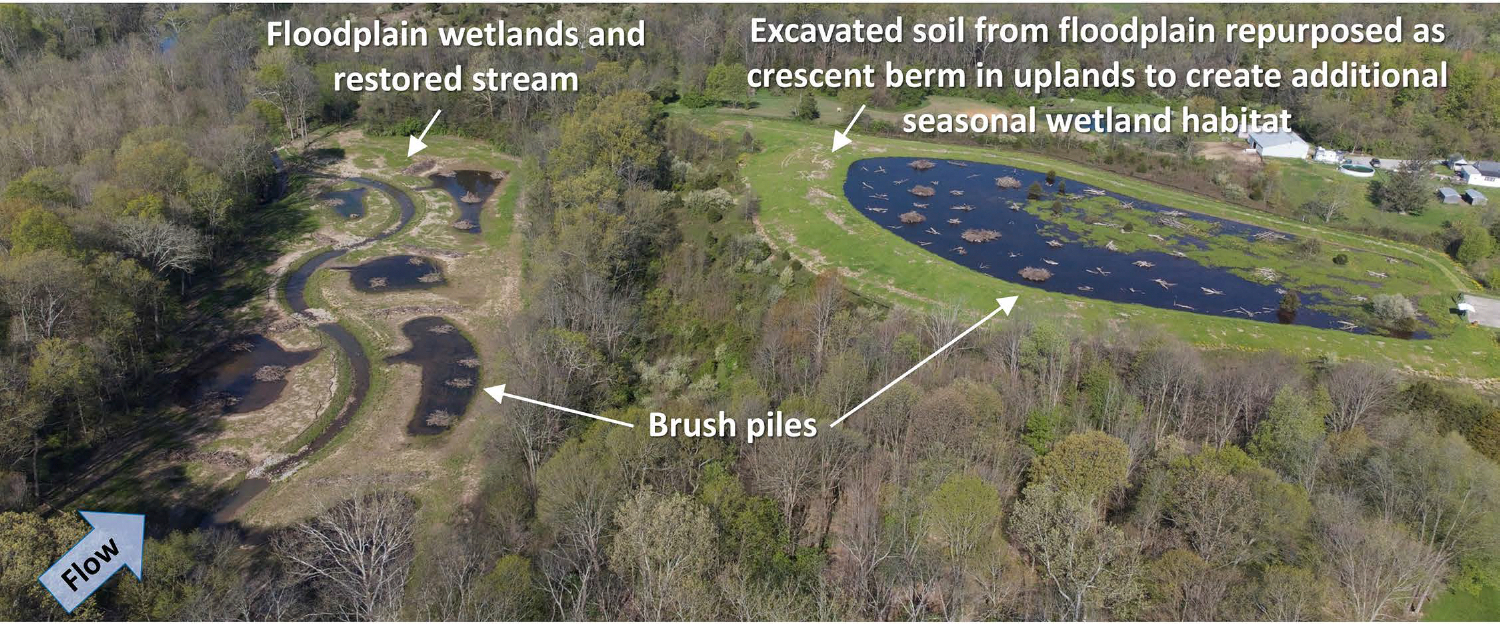
Aerial photo from spring 2024 of the East Fork Riparian stream restoration and wetland project less than one year following construction completion. Photo adapted with permission from aerial image by David Scheibenzuber, Clermont County Office of Public Information. https://doi.org/10.1371/journal.pwat.0000426.g004

Floodplain wetland projects can also be coupled with stream restoration. Taking a formerly channelized stream along the side of the valley and restoring a meandering stream across the valley bottom can not only restore a greater amount of habitat types, but in some cases can be more economical than a wetland restoration project that leaves the stream in an entrenched and enlarged condition. By restoring a smaller stream channel, less net excavation is required in the adjacent floodplain to connect the wetlands at a desired discharge rate. Furthermore, the excavated soil from the floodplain can be repurposed in the uplands to build additional wetland habitat features nearby. The East Fork Riparian wetland project was coupled with stream restoration on an unnamed tributary to the East Fork of the Little Miami River in southwest Ohio with a construction budget of ca. $350,000. Approximately 11,000 m^3^ of excavated soil from the floodplain created ca. 0.3 hectares of floodplain wetland habitat along with ca. 300 m of re-meandered (un-channelized) stream in a recently decommissioned agricultural field. The connections between the six sink-style wetlands and the restored stream are just 15 cm above the baseflow elevation in the stream, corresponding to a discharge of ca. 4% to 5% of the 2-year peak discharge (*Q*_2_) at which offloading flow into the wetlands begins. Additionally, the restored floodplain elevation is just 45 cm above the stream baseflow elevation, coinciding with a discharge of ca. 30% of the *Q*_2_, suggesting that the entire floodplain would have relatively frequent access for larger “flow through” events and may support the gradual expansion of floodplain wetland acreage depending on saturation frequencies and durations. Furthermore, the excavated soil from the floodplain was repurposed in the uplands to shape an elevated berm that created an additional ca. 0.9 hectares of seasonal wetland habitat supportive of amphibians. The berm also forms an elevated walking trail offering scenic views and a dry hiking path for users of the nature preserve (Fig 4). **Expanded habitat** is the sixth success criterion for floodplain wetland projects.

## Success criteria – economic efficiency and project durability

Success criteria related to the economic efficiency and project durability of floodplain wetland projects are gleaned from literature and experience, and arranged into the following topics: site location, valley setting, multi-stage treatment, stable river connections, site stability, construction sequencing, vegetation reestablishment, and community engagement. The corresponding success criteria related to economic efficiency and project durability reflect a synthesis of some of these topics (Box 1).

### Site location

Site selection can be extremely influential on a project’s economic efficiency and durability. Locating wetland restoration projects in floodplains can be an economically effective strategy in that every bucket of excavation creates additional space for water, as opposed to sites that require large amounts of excavation or stormwater piping to receive flows via gravity (i.e., without pumping). Open land use and non-forested land cover is also ideal in that the added costs and environmental impacts of tree removal are avoided. Additionally, avoiding sites with existing wetlands helps to streamline permitting costs and potential delays; however, there can also be value in conducting wetland restoration activities within existing wetlands that are low quality, uniform, or poorly connected hydrologically. Selecting project sites that **minimize extraneous costs** is our seventh overall success criterion and first criterion related to economic efficiency and project durability.

Additionally, having a nearby site that is outside of the floodplain to place the excavated soil can help control cost. In some cases, the excavated soil can be used to create additional habitat on the project site, such as seasonal upland wetlands that are inaccessible to fish and also provide spring habitat for amphibians and birds [58] (Fig 4).

Alternatively, finding contractors, landscapers, farmers, or community gardeners who want to reuse the soil for another purpose can provide large cost savings. The contractor on the Tipp City project had a use for the high-quality soil on a separate “rails to trails” project to help facilitate revegetation along the railroad berm that was being converted into a bike trail. Based on the competitive number of bids for excavated soil, the Tipp City Project enjoyed ca. 4- to 5-fold cost savings compared to reusing the soil onsite, and ca. 40- to 80-fold cost savings compared to projects that must pay a contractor to haul off the soil with no planned reuse. Conversely, a site with contaminated sediment that requires special disposal would likely be orders of magnitude more expensive than a site with soils suitable for reuse. Practitioners are encouraged to research the history of the site and watershed as it relates to the potential for contaminated sediment and use good judgment regarding the need for potential contaminant testing. **Minimizing soil haul-off costs** is proposed as our eighth success criterion.

### Valley setting

Flatter rivers with broad floodplains have different design constraints than steeper streams in more confined valleys. The desired offloading flow depth, connection constraints, and other project goals can all be factors in optimizing a design to a given site. In broad floodplains along flat rivers, the design and connection(s) can be relatively straightforward. But in a steep setting, a longer connection channel may be necessary to maximize the storage volume within the floodplain area while also offloading flows at a desired depth. At the YMCA project (Table 1) the bedrock elevation at the proposed floodplain wetland location was less than 0.4 m below the flow depth associated with the *Q*_critical_. Rather than taking the extremely expensive approach of excavating or blasting through bedrock to create a wetland sink, the project took advantage of the relatively steep slope of the river. A ca. 100-m connector channel was constructed to offload flows at the same *Q*_critical_ depth but at an upstream elevation that was 1 m above the bedrock elevation at the floodplain wetland location (see construction drawings (S1 Drawings in S3 File) in Supplemental Materials for additional details). In sum, the relatively small amount of excavation used to create the connector channel approximately tripled the effective sink volume in the floodplain wetland while avoiding the need to excavate through bedrock, providing another example of minimizing extraneous costs in the context of the valley setting of the project.

### Multi-stage treatment

Multi-stage designs are one way to increase the economic efficiency of water quality benefits of floodplain wetlands while minimizing extraneous costs in river valleys that experience an array of inundation depths and frequencies. Water quality benefits in floodplain wetlands can occur via sinks or flow-through processes. The advantage of sink features is that the prolonged residence time results in more effective sediment and pollutant removal and enhances the assimilation of inorganic nutrient species (Fig 2). The disadvantage of sink features is that they can only treat the water that is trapped in them, which in many cases is not a large portion of the total hydrograph volume for a given event (e.g., Fig 1).

By contrast, flow-through processes have the potential to treat a much greater portion of the hydrograph than sink features, but their lower residence times would likely lead to lower pollutant removal efficiencies. One way to combine the benefits of sinks and flow-through features is to create sinks that also serve as flow-through features during higher flow events (e.g., Table 1; Fig 4). Shallow-deep-shallow-deep sequences during high flow events or other multistage design features (e.g., Fig 2E) can help create contact between vegetated and reactive bank and bed surfaces and shallow/slow water flow-through at multiple flood stages. With deep areas sequenced into the flow paths, they can promote additional depositional opportunities even after the initial filling period (e.g., during events that exceed the conveyance capacity of the stream channel, when flood waters reach the floodplain surface and can flow over sinks and other deep/depositional features).

### Stable river connections

The connection between the river and the floodplain wetland typically requires special consideration and engineering designs to minimize erosion risks. Erosion at the connection could range from the relatively minor generation of additional sediment loads to cutting a new channel into the wetland that becomes the dominant flow path of the river (similar to the natural cutoff process that is common in meandering rivers [59]). Ensuring a **stable river connection(s)** is therefore proposed as the ninth success criterion for floodplain wetland restoration projects.

**Fig 5.**
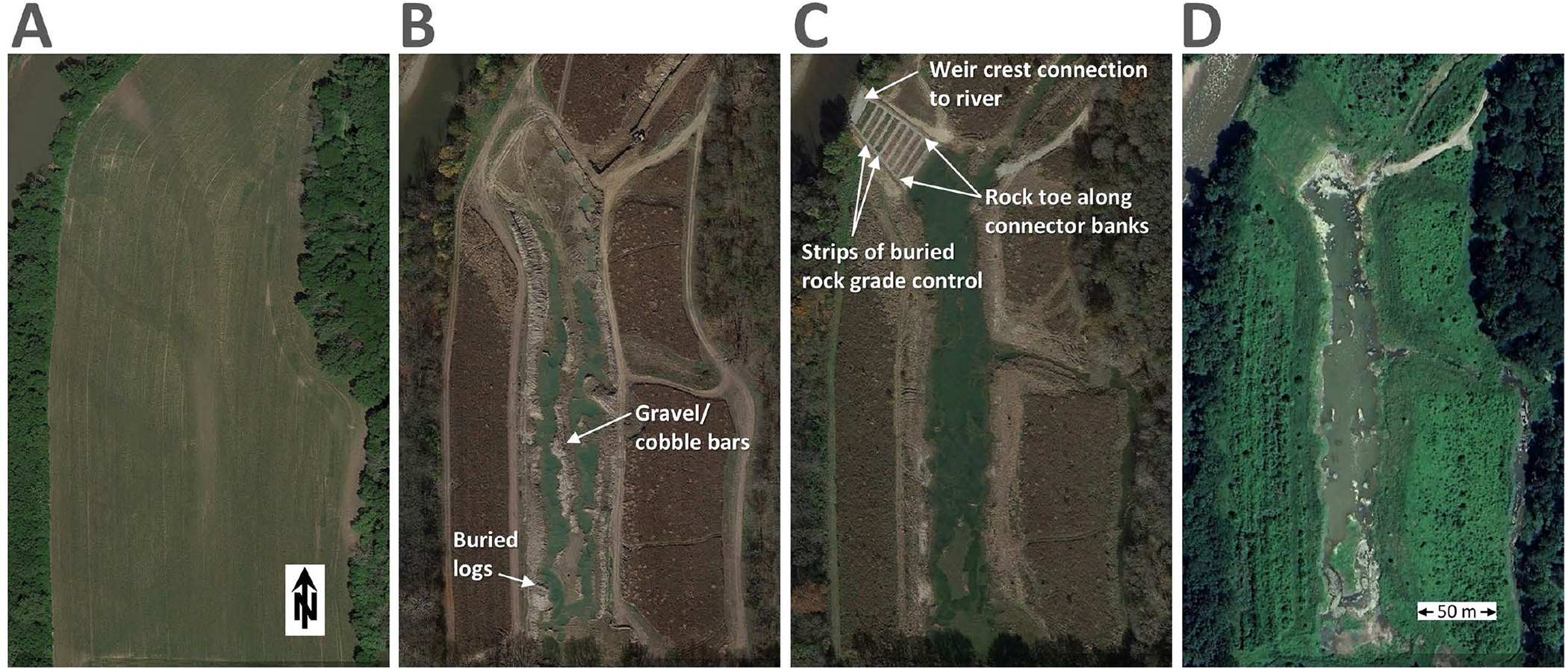
**Google Earth imagery of the Duke Park site documents the progression from** A) an agricultural field (6/13/2014); to B) a wetland sink excavation prior to creating a river connection (11/6/2020); to C) the constructed connection with buried rock grade control every 0.3 vertical meters and rock toe along the connection channel (11/5/2021), and finally, D) vegetation colonization of the rock in the connection channel (6/27/2024). https://doi.org/10.1371/journal.pwat.0000426.g005

Depending on the power of the river and the geomorphic setting, an array of engineering alternatives could be employed to ensure a stable connection such as rock chutes [27], rock toe, using live tree stakes, burying rock vanes to provide grade control, and tree vanes, among other strategies. We recommend designing connections for stability for up to and including the 100-yr discharge with a factor of safety that is agreeable to the project stakeholders (e.g., ~ 25–50%) [44]. In some cases, the rising limb of the stream hydrograph will be the critical design variable—i.e., what is the maximum offloading rate the connection is likely to experience when the wetland is empty and the water surface slopes entering the wetland are the steepest? Even in cases where shear stresses are less than the erosion threshold for good stands of native herbaceous cover (i.e., ~ 50–100 N/m^2^; ~ 1–2 pounds/ft^2^) [60], we have greater confidence when the armoring strategy for the connection uses a buried rock grade control every 0.3 vertical meters and rock toe in the connection channel as a means to protect the site during vegetation establishment (Fig 5C). Particularly in humid climates, the rapid growth of vegetation can quickly mask the presence of subtle rock features within a couple of growing seasons (Northwest portion of Fig 5D).

**Fig 6.**
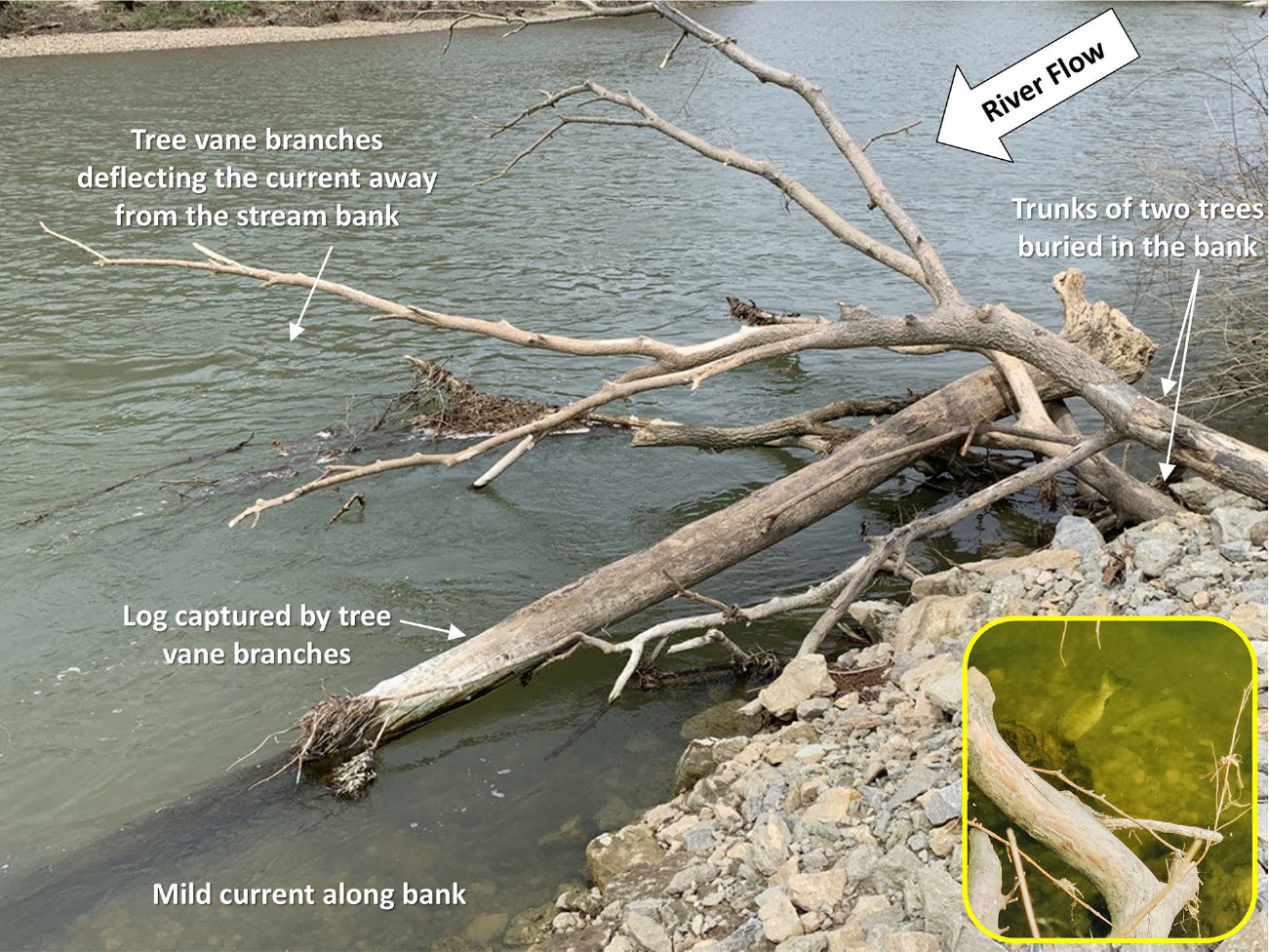
Photo of a tree vane purposefully buried in a streambank near the river/wetland connection to deflect flow energy away from the bank. Inset photo is smallmouth bass (*Micropterus dolomieu*) utilizing the habitat provided by the vane. Tree vane photo by R.J. Hawley, with inset photo by D. Knight. https://doi.org/10.1371/journal.pwat.0000426.g006

Another risk to floodplain wetland connections is erosion and migration of the main channel that may impact the wetland or flank the connection. Tree vanes have become one of our most frequently used strategies to protect against future migration of the main channel without large expansions in the footprint of the project. As compared to several hundred meters of boulder toe along an unstable outer bank. If a tree needs to be removed as a part of the wetland project, it can be reused as a flow deflector to keep the energy of the main channel directed toward the center of the river and away from an outer bank. The tree trunk is buried in the streambank with a depth of soil cover (typically greater than 1- to 2-times the tree diameter) and length (typically ~67–75% of the total length) that is guided by the “Large Wood Structure Stability Analysis Tool” [28] (url: https://www.engr.colostate.edu/~bbledsoe/streamtools/index.html). The treetop and associated branches protrude into the river to provide roughness in the water column and deflect the current away from the bank, mimicking the hydraulics associated with natural wood recruitment processes such as tree falls along undercut banks. The branches can also trap other large pieces of wood that float in from upstream, reinforcing the flow deflection benefits and helping to induce depositional zones along the toe of the bank that could provide a surface for natural reforestation and a more durable source of flow roughness and flow deflection (Fig 6). The scouring action induced by the ends of the branches can also facilitate clean gravel for nesting habitats for sport fish such as smallmouth bass (*Micropterus dolomieu*) that are of interest to some stakeholders.

### Site stability - preventing erosion & planning for deposition

In addition to stable river connections, good design strategies prevent erosion as well as provide for intentional areas for deposition. Excess floodplain erosion can degrade habitat and water quality and, in the worst cases, lead to cutoffs of the primary river channel which would likely be received as a detriment by numerous stakeholder groups [44]. By contrast, large amounts of sedimentation, particularly at the river-wetland connection could reduce the frequency that the wetland receives flows from the river. Sediment accumulation in the zone between the typical water table and floodplain elevations also reduces the effective volume of the wetland sink [61]. As such, **minimizing floodplain erosion** and **accommodating sedimentation** are the tenth and eleventh success criteria for evaluating floodplain wetland restoration projects.

Most floodplain sites can be adequately stabilized with good stands of native herbaceous cover [44,60]. Strategically incorporating large wood and brush piles in the floodplain wetlands (Fig 4) adds hydraulic roughness, which acts to dissipate erosive energy and help protect the site during vegetation establishment [44]. The wood also provides a carbon source that can help facilitate desired nutrient and pollutant transformations [52].

**Fig 7.**
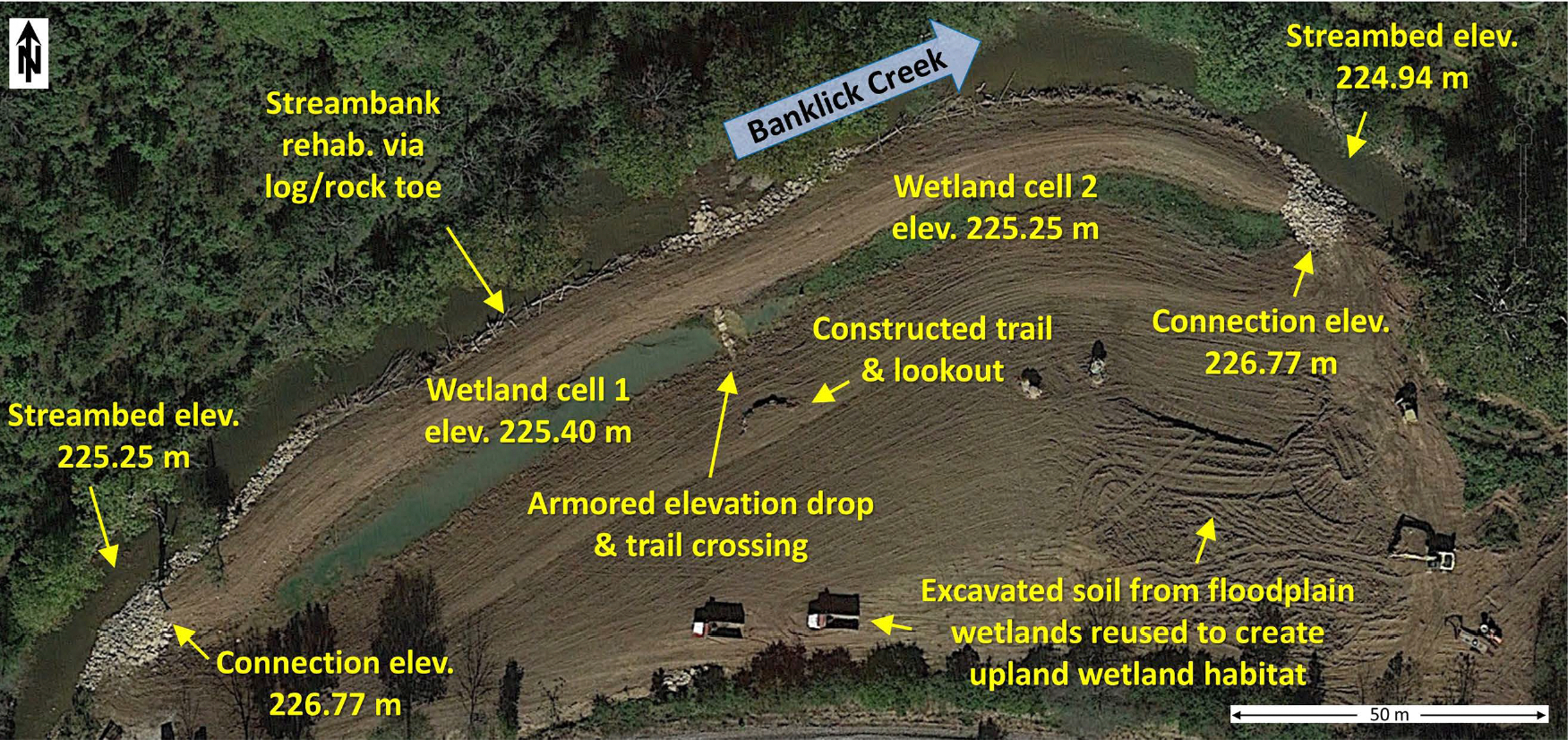
The Wolsing Woods floodplain wetland is a combination flow-through and sink design in a relatively narrow and steep valley. Text overlying a 10/17/2018 aerial photograph (adapted from Google Earth) indicates the upstream (left side of image) and downstream (right side) connections, two wetland cells separated by the armored elevation drop, and the reuse of the excavated floodplain soil to create additional upland wetland habitat. https://doi.org/10.1371/journal.pwat.0000426.g007

In steeper and more confined valleys, rock structures may be required to effectively dissipate concentrated areas of erosive energy. In a flow-through system in a relatively steep valley, one grade control option is a constructed riffle that drops the grade over a narrow area of rock armoring between two otherwise flat wetland cells. The Wolsing Woods Project provides a good example of this, using boulder steppingstones to create the grade-controlling elevation drop that also serves as a trail crossing (Fig 7). When sizing rock armoring for a grade controlling structure such as a constructed riffle, the Khan and Amad equation [62] has stood the test of numerous of our constructed applications and its predictive performance is consistent with the findings of others [63].

It is also critical to plan for deposition within floodplain wetlands. Similar to stormwater control measures designs that incorporate sediment forebays, the functional lifespan of floodplain wetlands can be extended by providing “wintering holes” and other deep features near the wetland entrance that provide intentional locations for sediment to accumulate but can also serve as habitat for fish. In our experience in southwest Ohio and northern Kentucky, the wildlife and recreational value of these sediment sinks can be expanded by making them at least ~1.3 meters deeper than the typical winter pool elevation to offer fish “over-wintering” habitat. Depending on the available space and other goals, the pools can be constructed to provide intentional off-channel nursery, spawning, and other habitat types by incorporating large wood, gravel/cobble bars (e.g., Fig 5B), or other features. Costs can be controlled by reusing alluvial cobbles/gravels, wood, and brush encountered during the wetland excavation. We recommend consulting with a fish biologist to tailor designs to a given site or species of interest.

In extremely flat systems that are primarily tailored to improve water quality or reduce nutrient loads, the connection and associated sediment forebay may need to be designed to scour sediment from the connection between the river and wetland, as well as provide maintenance access for equipment to excavate accumulated soil. As opposed to higher energy sites where wider and flatter connections are more appropriate, a low-energy wetland setting may require customization of broad-crested weirs based on design examples reported in the literature [35,64,65]. A narrower, steeper weir that prevents deposition at the connection could be necessary to maintain the desired hydrologic connectivity between the wetland and the river. In such low-energy wetland designs, the sediment forebay at the connection would be expected to have relatively high rates of sediment accumulation. To the extent feasible within the confines of the site, the size of the sediment forebay/wintering pool should be maximized to ensure sediment capture while also addressing other stakeholder goals. Depending on the community goals of the site, the accumulated sediment may provide a sustainable supply of nutrient-rich soil that could complement a community composting or topsoil program. Alternatively, a site with no maintenance budget for sediment removal from a sediment forebay may need to be designed with long-term functionality in mind. By over-excavating the basin to be deeper than the typical winter pool elevation, it may prolong the functional life of the wetland. In this way, the wetland could be constructed as open water/ emergent habitat, and slowly transition to more of a scrub/shrub and forested wetland as sediment gradually accumulates within the wetland. Although sediment accumulation rates will vary based on the supply of sediment, frequency of inundation, etc., one regional analysis estimated that a 1.5-m deep wetland would fill in with sediment between one and five centuries [23].

### Construction sequencing

One of the best practices to control cost and prevent difficult construction challenges is to use strategic construction sequencing. Conducting excavation activities in the floodplain when the water table is low helps to avoid large costs associated with dewatering, building elevated or armored haul roads, and delays associated with equipment becoming mired. In the humid temperate setting of southwest Ohio and northern Kentucky, low water tables typically occur in late summer and early autumn. Starting the excavation at the farthest location from the construction access point and systematically working the excavation back towards the site entrance enables the construction equipment to stay on the dry floodplain surface as opposed to risking getting stuck by excavating or hauling within the constructed sinks.

Secondly, it is critical to conduct the bulk of the floodplain excavation prior to creating an active connection to the river (Fig 5B). The obvious solution is to excavate as much of the floodplain as practicable leaving only a small but functional plug by the future river connections. This practice avoids the expense and nuisance of conducting excavation and site stabilization practices in the active flow. Hypothetically, using straw mat or coir fabric across the entire site on the Rainbow Run would have added ca. $100,000 to the project, nearly doubling the total construction cost. Proportional cost increases would be expected for all the projects listed in Table 1 (e.g., straw mat and coir fabric typically range ca. $5–15 per square yard installed, scaling to costs on the order of ca. $100,000 per hectare). By contrast, good construction sequencing can facilitate the establishment of inexpensive temporary seeding such as annual rye/oats to provide a good stand of covering vegetation prior to creating a connection to the river. The temporary cover not only helps to protect against erosion and limits the need for expensive coir fabric or straw matting, but also helps to protect the site from rapid colonization by weeds, invasive plants, or otherwise undesirable vegetation.

### Vegetation reestablishment

An important component to long-term site stability is the establishment of protective vegetation [66]. Good stands of native herbaceous cover provide wildlife values and protect the site from erosion [67], while trees and shrubs can provide even deeper roots and bank stability [68], increased shade, and a future carbon source for functions such as denitrification. As mentioned above, poor construction sequencing can substantially increase revegetation costs and decrease economic efficiency. Therefore, **minimizing revegetation costs** is proposed as the twelfth success criterion.

Temporary herbaceous cover can provide initial stabilization and cover for the establishment of a more permanent herbaceous layer, followed by reforestation that may offer more durable erosion control, habitat, and other benefits. In most floodplains, wetland excavation activities are likely to uncover a native seedbank of herbaceous plants [69] such that a site may need a limited amount of outside seeding other than the temporary cover. Almost immediately after construction at the Tipp City wetland, numerous obligate native wetland plants that were not planted via plugs or a seed mix were abundant across the site (e.g., Canadian waterweed (*Elodea canadensis* Michx.), coon’s tail (*Ceratophyllum demersum*) longleaf pondweed (*Potamogeton nodosus*), and sago pondweed (*Stuckenia pectinata*)). Taking advantage of the formerly buried seedbank not only helps to control the costs and labor of planting wetland plugs, but also reminds us that these floodplain environments were likely supportive of wetland habitats prior to European settlement and the associated accumulation of alluvial sediments.

Reforestation costs can also be minimized by establishing small stands of native obligate wetland trees such as black willow (*Salix nigra*) via live cuttings, i.e., live staking [70], of existing stands nearby or from previous wetland projects. After a few years of establishment, additional trees can be propagated via live cuttings from the stands on site. American sycamore (*Platanus occidentalis*), another common wetland tree, can readily propagate by seed dispersal from nearby sources. Additionally, more expensive nut trees can be effectively planted by squirrels if nearby populations exist. On sites that lack nearby stands of native trees, reforestation can be catalyzed by establishing small dense groves of native wetland hardwoods thereby limiting installation and maintenance costs and eventually becoming the source for natural recolonization of late-successional species [71].

### Community engagement

Planning, regulatory, and scientific communities increasingly acknowledge that genuine community engagement is not only more equitable for traditionally underserved communities [72] but can also lead to more sustainable and expansive environmental benefits in both the short and long term [73,74]. One potential barrier to wetland construction can be the perceived risks of creating “mosquito ponds.” Particularly in urban areas, the perceived risks of contributing to the spread of mosquito-borne diseases such as West Nile virus can be challenging for proposed wetland projects to overcome. A recent study of constructed stormwater wetlands, detention ponds, and retention ponds in central Ohio documented higher mosquito larvae abundance and diversity in constructed wetlands but fewer numbers of known disease vector species such as *Culex pipiens* and *Aedes vexans* compared to detention ponds that had lower abundance and diversity but a higher number of vector species [75]. Ohio Department of Health data on the Tipp City project documented a relatively balanced mosquito population, including just one individual of the *Aedes albopictus* mosquito that feeds during the daytime and no carriers of West Nile virus. By comparison, a residential property 2-km from the wetland project associated with a mosquito complaint initiated an Ohio Department of Health survey. Samples from outdoor plant saucers and watering cans had an abundance of both daytime mosquitos and *Culex pipiens*, including one sample that tested positive for West Nile virus. These data align with common guidance from health departments regarding eliminating outdoor vessels that hold water (e.g., plastic toys, old tires, etc.), regularly cleaning gutters, and changing water in pet dishes, watering trays, bird baths, etc. as they are common sources of mosquito breeding that lack the ecosystem defenses in wetland habitats such as dragonflies, fish, bats, etc. [76].

This incidence highlights the importance of outreach to ensure that the community’s concerns and preferences are heard and respected at the earliest opportunity, not as a top-down effort to encourage adoption of the project. Therefore, **ensuring community support** and maximizing the equitable delivery of benefits [72] is the thirteenth and final success criterion for floodplain wetland projects.

It cannot be overstated that it is critical to provide the community a place at the table as genuine co-managers of waterway management projects. An economically disadvantaged community may have chronic flood problems and may also lack recreational areas and public green spaces. In this case, it may be more beneficial and equitable to design a flow-through system in the floodplain as a green space that drains following flood events and can provide an array of recreational uses during most of the year. In such a system, a flow-through “high flow channel,” floodplain bench, or “green sink” with a drainage system may provide similar opportunities for water quality improvement and flood reduction as a floodplain wetland, while also facilitating the desired social uses of the space. While an uncompromising habitat goal of “wetland restoration” could be a dealbreaker for an otherwise beneficial project, a thoughtfully co-designed floodplain enhancement project that prioritizes community goals and maximizes the equitable delivery of benefits while meeting institutional values could provide a pathway toward implementation.

## Success criteria application

**Fig 8.**
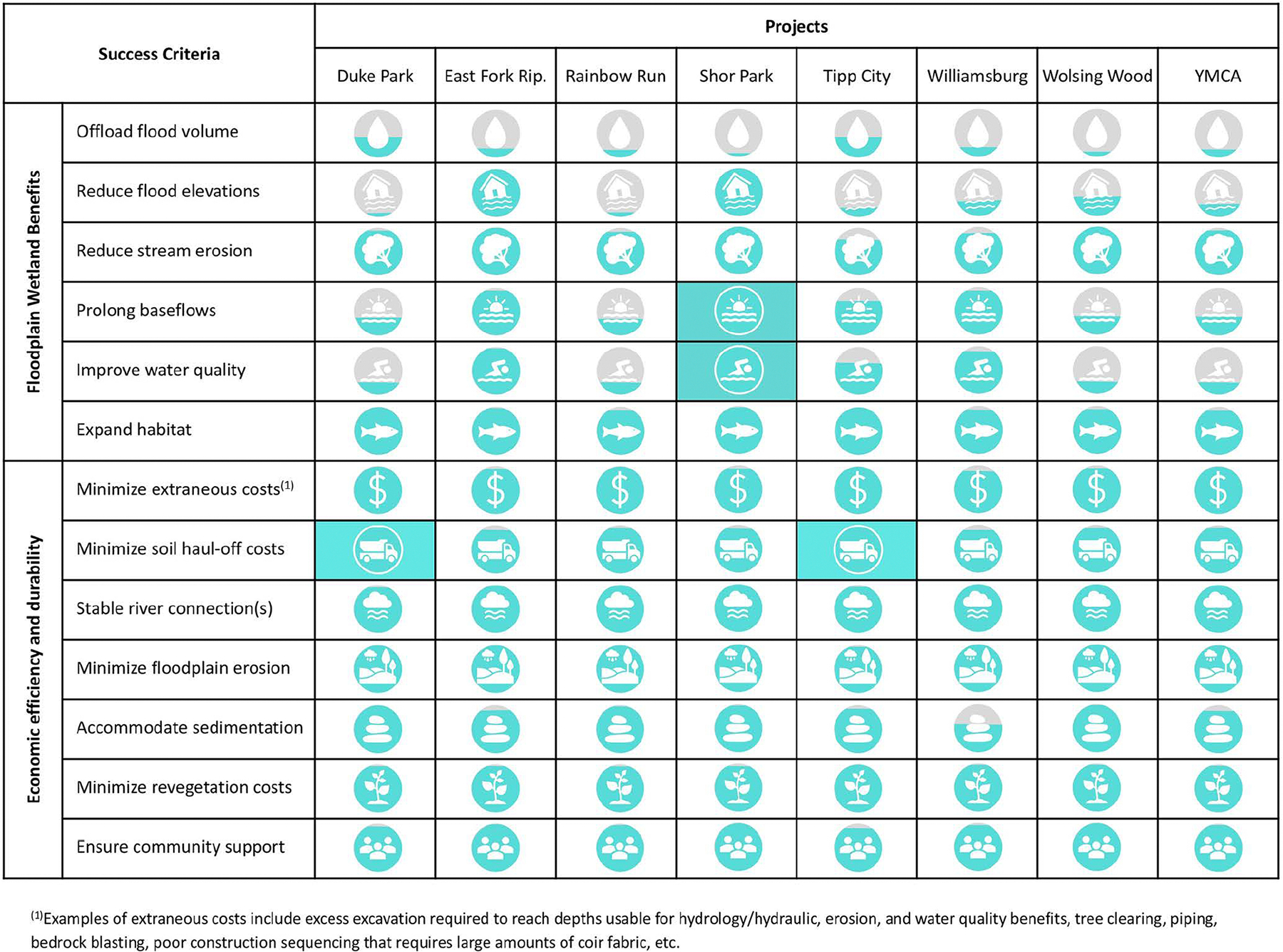
Comparison of eight (8) constructed floodplain wetland projects via the success criteria developed herein. Extent of shading within each circle represents the relative performance of the project for that success criterion compared to other projects and in the context of the overall river setting (formatting adapted from [72]). Completely shaded rectangles represent exceptionally high performance that would not be easily repeatable in dissimilar settings. https://doi.org/10.1371/journal.pwat.0000426.g008

Fig 8 shows how the success criteria can be used to compare the eight constructed floodplain wetland projects. The comparisons in Fig 8 represent relative comparisons of the success criteria between projects as opposed to absolute measures of project performance. Having developed the criteria from these previously constructed projects, the criteria could be used as absolute measures to evaluate performance in subsequent floodplain wetland projects.

Designs of the eight constructed projects were explicitly optimized to maximize specific stakeholder goals (Table 1), such that not every project included all types of features and scales of benefits. Some of the most inherent tradeoffs between competing benefits [77] are evident in Fig 8. Williamsburg was designed to maximize water quality improvements in a relatively steep and narrow valley for the relatively large East Fork of the Little Miami River. This resulted in a connection elevation that was relatively low and a floodplain wetland system that was extremely flat. Although the water quality performance has been encouraging, sedimentation at the inlet connection has required active removal to maintain the desired connection elevation to continue to offload flows during events that correspond to the majority of the annual runoff-generated in a typical year. A more concentrated and steeper connection weir could have helped to keep the sediment in suspension through the connection and concentrated more of the sedimentation in the wintering hole; however, the low energy and long residence time requirements to maximize water quality also inherently promote sedimentation.

Similar tradeoffs have been discussed throughout the manuscript. Projects with a higher connection elevation to keep the sink empty until nearing the *Q*_critical_ threshold maximize the offloading of erosive flows and increase the likelihood of maintaining streambed stability. E.g., in Fig 8, Duke Park performs better than Tipp City at reducing erosion but less well at improving water quality.

However, most success criteria are not mutually exclusive and can perform well across an array of design objectives and stakeholder goals (Fig 8). All the projects performed well regarding minimizing floodplain erosion and revegetation, soil haul off, and extraneous costs, as well as at ensuring community support and stable river connection(s). All the projects substantially expanded the habitat of the site compared to the pre-project conditions.

Two projects, Duke Park and Tipp City, were exceptional at minimizing soil haul-off costs by spreading the word about the quality of the soil on site and finding stakeholders who felt the soil was worth their effort to haul it off at no cost (shaded blocks in Fig 8). The stream daylighting project as Shor Park was exceptional at improving water quality and prolonging baseflow due to the ability to pool the water into the entire restored floodplain prior to discharging over the cascading channel that reconnected the daylighted channel to the receiving stream network. The elevation drop of the site also facilitated an infiltration-style outlet with an underdrain that prolonged the discharge of baseflow enough to support fish in the receiving stream that was an ephemeral flow class prior to the project.

These criteria were easy to apply as a post hoc comparison for these eight constructed projects. Not every criterion will be relevant or used the same way in all global settings. In a highly confined, “canyon-like” valley where the entire valley bottom is in the floodplain, “minimizing soil haul-off costs” may still be a worthwhile goal but the relative scale of the criterion would need to be adjusted for the setting. That is, all projects may need to haul the sediment out of the canyon but a project that found a nearby location to the canyon exit may score relatively better than a project that hauled the sediment hundreds of kilometers away from the canyon exit. One can envision such relative adjustments for “minimizing revegetation costs” for projects in more arid climates. Furthermore, the criterion of ensuring a “stable river connection” may not even be feasible (or desired) in a highly dynamic setting such as a braided river or an alluvial fan. Researchers and practitioners are encouraged to adjust and tailor these success criteria to accommodate both natural and community attributes of their setting.

Potentially the biggest value to practitioners could be incorporating these success criteria and lessons learned into the design of future floodplain wetlands on the front end. We would recommend the use of a two-dimensional model to assist with the design of the connection in a low-energy setting like Williamsburg to better balance the water quality goals with the goal of limiting deposition at the connection. On a highly visible site in an urban area, we would recommend community engagement efforts such as design workshops to inventory community priorities such that they could be incorporated into the planning and design to the extent feasible within the context of other funding criteria and site constraints. A floodplain sink that provides amenities such as soccer fields, walking paths, etc., may not expand wetland habitat but could more equitably deliver benefits of offloaded flood volume, reduced flood elevations, reduced stream erosion, and improved water quality that are comparable to those of a more natural floodplain wetland. The success criteria provided herein can help planners and designers optimize such objectives in the context of other community needs and preferences.

## Conclusions

A variety of sources suggest that numerous historical and contemporary societies have contributed to amplified rates of landscape erosion and sediment accumulation in riverine floodplains [4,5,12], reducing the available services that the floodplains can provide to modern societies. On eight constructed floodplain wetland projects, we have encountered up to ~1–3 meters of post-settlement alluvium covering historic alluvial layers that are abundant with seeds of obligate wetland plants native to North America. This underscores the vastness of historic floodplain wetlands, the scale of potential benefits they provided, and the relative simplicity of restoring those benefits: excavating post-settlement alluvium from the floodplain and relocating it or reusing it outside of the floodplain to seed new wetland ecosystems, restore compacted terrestrial systems, or other useful applications.

Modeling and analyses can be used to optimize designs to maximize stakeholder goals for a particular site. In the subcritical flow environments of many floodplains, flow-through wetland systems that expand the cross-sectional conveyance area are likely to lower the water surface elevation at the wetland site and have the potential to benefit reaches immediately upstream, depending on the river slope and the presence of dams, bridges, levees, and other hydraulic constrictions. Coupling floodplain wetland projects with levee setbacks [78] and the removal of other constrictions such as low head dams and undersized culverts/bridges could facilitate even greater reductions in flood elevations.

By contrast, reducing the frequency of streambed mobilizing events might be best optimized via floodplain sink designs that offload flows just prior to reaching the stage associated with the *Q*_critical_ for streambed mobilization. If the volume of the floodplain sink is comparable to the volume of the portion of the hydrograph that exceeds *Q*_critical_, the resulting hydrograph might no longer cause streambed mobility in the reach adjacent to the floodplain and reaches downstream depending on additional discharge inputs from tributaries and the hydrograph timing. Particularly in the case of disturbance-sensitive species such as macroinvertebrates [41] and early juvenile mussels [42], a prolonged period of seasonal streambed stability could be the difference between completing a vulnerable portion of their life cycle or experiencing a lethal disturbance [79,80].

Water quality benefits can be enhanced by providing multiple tiers of vegetated surfaces that stormwater can interact with. Deep-shallow sequences and a meandering flow path can further help to facilitate deposition and prolong residence times for nutrient assimilation across a gradient of flood stages. Water quality benefits can be increased by extended storage (sink designs) and slow-release features to remove pollutants and provide storage for the next weather event. By providing a connection stage that is as low as feasible for a given site and set of stakeholder goals, a floodplain wetland has the potential to treat more runoff-generating events throughout the year as opposed to being limited to relatively rare flood events.

The excavated surface elevations in the floodplain wetlands can be tailored to a variety of desired wetland habitats such as forested wetlands, emergent wetlands, or open water habitats based on the summer and winter groundwater levels (which are typically the baseflow elevations in the adjacent river). These elevations can be further refined by plant surveys of various elevations in the adjacent river (e.g., elevation of lowest trees, elevation of lowest aquatic plants, pools with no vegetation, etc.).

Lastly, we emphasize the importance of genuine community engagement in planning, co-designing, and implementing these projects [72,73]. There are numerous potential benefits to a variety of stakeholders (e.g., reduced flooding, improved water quality, fishing, birding, community greenspace, trails, recreational fields, a source of topsoil, etc.) such that a floodplain wetland project can be optimized for any number of community priorities. In this light, it is important for ecosystem restoration advocates to avoid letting the idyllic goal of “habitat restoration” be a barrier to the broader ecosystem and societal benefits of removing post-settlement alluvium from floodplains. The strategies outlined herein would apply to any number of floodplain enhancement projects that promote and restore floodplain functions [25], even those that do not explicitly restore “wetland” habitats. Although the constructed projects examples are concentrated in North America, the history of watershed deforestation and sediment accumulation in floodplains across the world underscore the potential relevance of the approach to other regions. We hope that future international research expands and improves this approach in other regions.

## Supplementary Material

S4 File - S1 Report

S3 File - S1 Drawings

S2 File - S2 Data

S5 File - S2 Report

S1 File - S1 Data

## Figures and Tables

**Table 1.** Location, size, features, primary and secondary design objectives, and cost data on eight (8) constructed floodplain wetland projects. “Flow through design” implies a wetland with both an upstream and downstream connection to the river, “sinks/depressions” indicate that the topography of the floodplain wetland can detain water after being filled, “extended discharge via drainage system” implies that the project had an outlet control feature such as an underdrain with a restricted outlet that prolonged the release of stored water from the wetland, and “coupled with stream restoration” means that the project incorporated both floodplain wetlands and stream restoration.

Project Name, adjacent stream/river, city and state	Latitude, Longitude	Approx. wetland area (hectares)	Const. Year	Const. cost	Flow through design (yes/no)	Sinks/ depressions (yes/no)	Extended discharge via drainage system (yes/no)	Coupled with stream restoration (yes/no)	Primary design objective	Secondary design objective	Ancillary benefits
Duke Park, Spring Creek & Great Miami River, Troy, OH	40.066168°, −84.212123°	3.1	2020	$422,172	N	Y	N	N	reduced erosion	nutrient assimilation	habitat expansion, topsoil source, fishing
East Fork Riparian, East Fork Little Miami River, Lynchburg, OH	39.232620°, −83.832981°	1.2	2023	$353,901	N	Y	N	Y	nutrient assimilation	habitat expansion	flood attenuation, reduced erosion, trails
Rainbow Run, tributary to Little Miami River, Clifton, OH	39.816981°, −83.812846°	1.0	2023	$111,280	N	Y	N	N	nutrient assimilation	habitat expansion	reduced erosion, birding
Shor Park, unnamed tributary to East Fork Little Miami River, Milford, OH	39.109450°, −84.240253°	0.05	2021	$111,980	N	Y	Y	Y	habitat expansion	enhance baseflow	nutrient assimilation, reduced erosion, trails
Tipp City, Great Miami River, Tipp City, OH	39.958960°, −84.143902°	2.9	2022	$254,017	N	Y	N	N	nutrient assimilation	habitat expansion	reduced erosion, topsoil source, fishing
Williamsburg, East Fork Little Miami River, Williamsburg, OH	39.064912°, −84.053465°	1.5	2022	$508,177	Y	Y	Y	N	nutrient assimilation	habitat expansion	flood attenuation, prolonged baseflow
Wolsing Woods, Banklick Creek, Independence, KY	38.955876°, −84.573132°	0.2	2018	$191,414	Y	Y	N	Y	reduced erosion	habitat expansion	flood attenuation, trails
YMCA, Gunpowder Creek, Burlington, KY	39.000738°, −84.725136°	0.70	2015	$129,943	N	Y	N	N	reduced erosion	habitat expansion	nutrient assimilation, trails, birding

https://doi.org/10.1371/journal.pwat.0000426.t001

## Data Availability

All data are in the manuscript and supporting information files.

## Box 1. Floodplain wetland planning, design, and construction highlights.

- Select sites that minimize extraneous costs:
  - open floodplains with limited tree clearing requirements or other factors that use construction funding for tasks other than wetland excavation and
  - leverage site topography to minimize excavation activities that do not contribute to wetland and other features desired by stakeholders.
- Repurpose the excavated floodplain soil:
  - reuse by landscapers, developers, farmers, community gardeners, etc., or
  - a nearby location outside the floodplain to create other features such as additional wetland habitats, elevated trails, or other recreational features.
- Strategically sequence construction:
  - keep construction equipment on dry surfaces and
  - establish herbaceous cover prior to constructing the connection to the river.
- Minimize revegetation expenses:
  - capitalize on the native seedbank that may be buried under post-settlement alluvium and
  - low-cost reforestation strategies such as live stakes harvested from on-site sources, establishing hardwood groves that can support future propagation to adjacent areas, and capitalizing on natural propagation by nearby tree populations.
