# Supplementary material for: Success criteria comparison of eight implemented projects to improve the planning, design, and construction of floodplain wetlands
Source: PLOS Water. Author manuscript; Available in PMC 2026 Jun 16. (PMC13266620; doi:10.1371/journal.pwat.0000426)
Supplement: S4 File - S1 Report [file NIHMS2157087-supplement-S4_File_-_S1_Report.pdf]

Floodplain Habitat Restoration Project – Great Miami River Hydrologic/Hydraulic Analysis Memorandum

Sustainable Streams LLC
Science · Service · Solutions

Prepared for United States Department of Fish and Wildlife and the Miami County Park District
By Sustainable Streams
February 2019

This memorandum documents the hydraulic modeling results for the proposed floodplain habitat restoration project adjacent to the Great Miami River, near Duke Park in Troy, Ohio (Figure 1). This reach is located within the Floodway (Zone AE), within which no substantial increase to the 1% annual chance flood elevation is allowed. The goal of this memorandum is to provide support for the applications to permitting authorities, such as the US Army Corps of Engineers (USACE) and the Miami Conservancy District (MCD), with the objective of illustrating that the proposed mitigation efforts will result in an average decrease in the 100-year flood elevation of approximately 0.02' from the downstream end of the project's proposed disturbance limits through the upstream limit of the effect of the project as compared to the Federal Emergency Management Agency's (FEMA) preliminary revised Flood Insurance Study (FIS), dated October 29, 2018. This project aims to establish a lower and more frequently inundated floodplain for a portion of the Great Miami River.

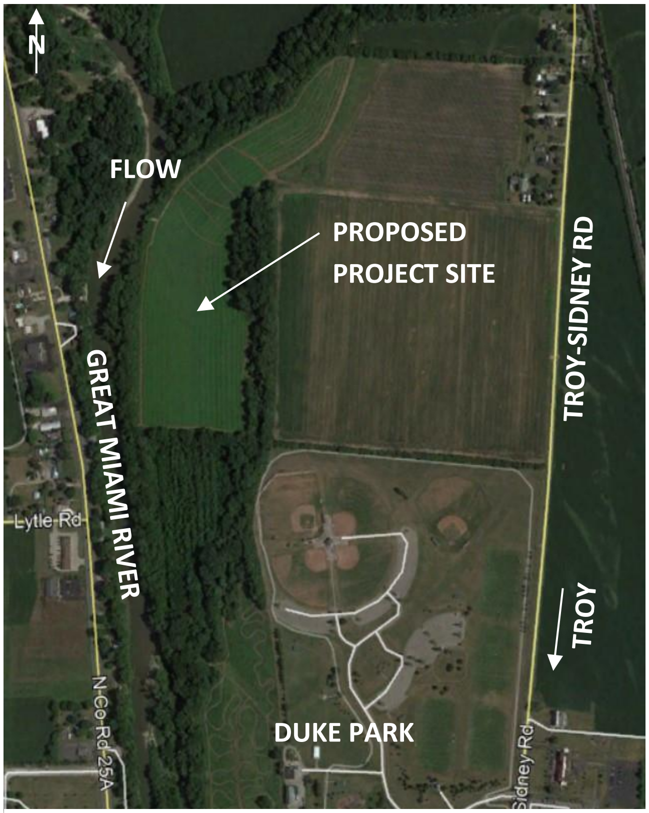

Figure 1 – The project is located on the east bank of the Great Miami River, just north and west of the baseball field complex on the north end of Duke Park in Troy, Ohio. Aerial image from Google Earth.

Project Background

The primary goals of this project are to create a basin in the floodplain of the Great Miami River that will have a lower connection to the river than the current condition, providing wetland habitat and wintering holes for sensitive species, reducing flood elevations, and offloading flow near the crests of seasonal peak flow hydrographs for which field indicators suggest disturbance of a majority of the streambed particles.

The proposed mitigation strategies include excavation of an overflow connection optimized to offload flows that would otherwise contribute to streambed disturbance. The offloaded volume will flow into a ~20 ac-ft storage basin that will provide flood storage and wetland habitat. Within the basin will be multiple wintering holes that will provide seasonal refugia for rare fishes.

Descriptions of Hydraulic Models

Hydrologic and hydraulic models of the Great Miami River and its tributaries in Miami County, Ohio were previously developed by FEMA as a part of the preliminary revised FIS, dated October 29, 2018. These models, provided by FEMA to Sustainable Streams in HEC-RAS format, were used as the baseline for the creation of the proposed project's hydraulic model. Prior to creating the proposed model, Sustainable Streams performed a channel and floodplain survey at the project site. Surveyed channel and floodplain transects, located between river stations 799+00 and 840+00, were imported into the FEMA HEC-RAS model to create increased precision for the immediate reach surrounding the proposed project. This revised model, containing both the FEMA and imported surveyed transects, would represent the existing conditions to which the proposed conditions would be compared. It should be noted that the increased precision of the existing model resulted in some changes to the existing water surface profile as compared to the FEMA FIS (e.g. +/- 0.18 ft). Finally, a proposed model was created by generating revised transects from a proposed grading plan, developed in AutoCAD. These revised transects are located between river stations 816+00 and 830+00.

For the purposed of flood elevation evaluation, the 1% discharges provided by FEMA were used (e.g. 23,600 cfs in the reach adjacent to the work).

The models indicated that as a result of the proposed project, the 100-Yr water surface elevation (WSE) would drop an average of 0.02' through the project reach and extending up to the upstream limit of the project's effect on the WSE. See Figures 2, 3 and Table 1 for comparisons of the WSEs associated with the existing and proposed models. Even though the floodplain excavation was limited to ~1,400 ft (Sta 816+00 to 830+00), the benefits to the flood elevations extended nearly 10,000 ft upstream according to the models. The maximum drop on the water surface elevation (0.07 ft) occurs at the upstream end of the wetland, near the connection to the Great Miami River (Figure 3).

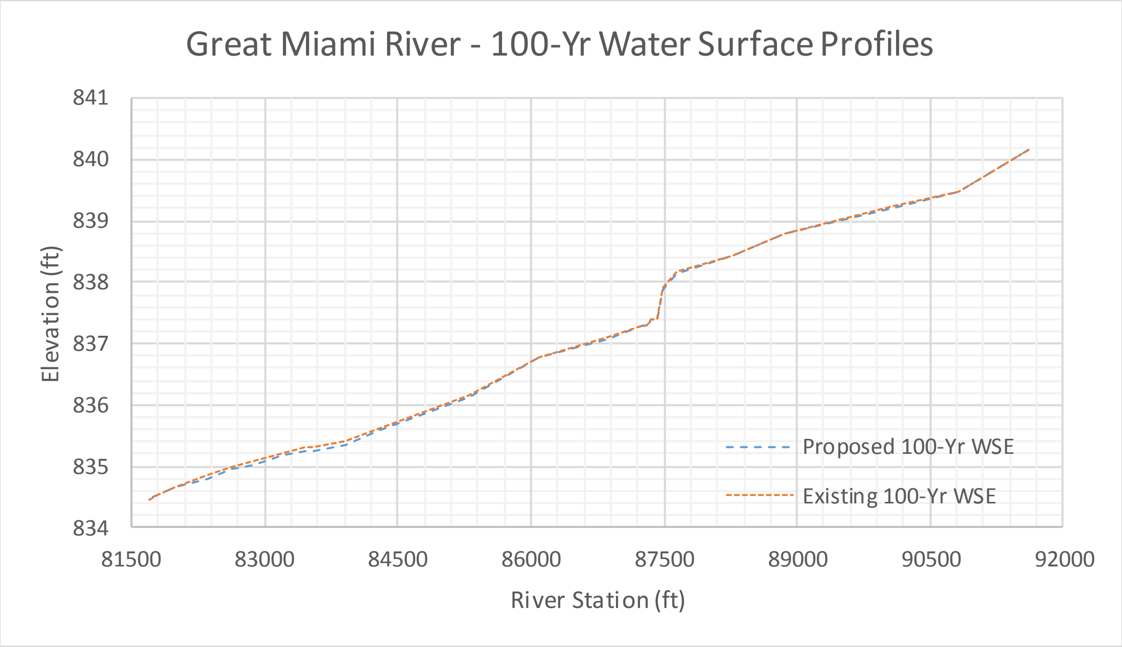

Figure 2 - Estimated 100-Year water surface elevations associated with the existing and proposed conditions.

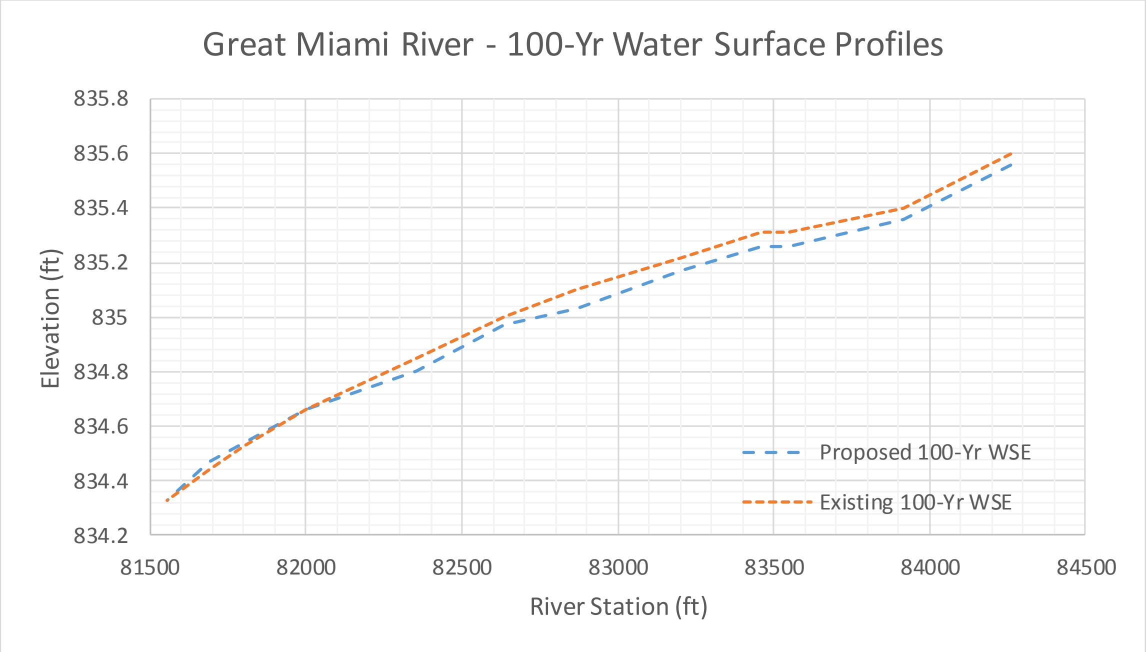

Figure 3 - Estimated 100-Year water surface elevations associated with the existing and proposed conditions, shown for the reach with the greatest benefit. The proposed floodplain excavation occurs between stations 816+00 and 830+00.

Table 1 - Predicted change in water surface elevations associated with the proposed restoration efforts. The calculated weighted average is a 0.02' decrease in 100-Year WSE from the downstream limits of project's disturbance limits through the upstream limit of the effect of the project.

River Station *ft*	Existing 100-Yr WSE FEMA Model *ft*	Existing 100-Yr WSE FEMA + Surveyed Transects *ft*	Proposed 100-Yr WSE *ft*	Change in WSE *ft*	
91612	840.13	840.15	840.15	0.00	
90814	839.43	839.47	839.46	-0.01	
90096	839.20	839.24	839.23	-0.01	
88868	838.75	838.80	838.79	-0.01	
88276	838.39	838.45	838.44	-0.01	
87657	838.10	838.16	838.15	-0.01	
87493	837.84	837.91	837.89	-0.02	
87426	837.31	837.40	837.37	-0.03	
87391	837.33	837.41	837.39	-0.02	
87354	837.32	837.40	837.38	-0.02	
87304	837.19	837.29	837.26	-0.03	
87258	837.22	837.31	837.29	-0.02	
86863	837.00	837.10	837.08	-0.02	
86093	836.68	836.79	836.77	-0.02	
85311	836.03	836.17	836.14	-0.03	
84259	835.42	835.60	835.56	-0.04	
83915	-	835.40	835.36	-0.04	
83550	-	835.31	835.26	-0.05	
83461	-	835.31	835.26	-0.05	
83204	835.09	835.22	835.17	-0.05	
82866	-	835.10	835.03	-0.07	Connection Station
82638	-	835.00	834.97	-0.03	
82356	834.76	834.85	834.80	-0.05	Excavation Zone
81999	-	834.66	834.66	0.00	
81800	-	834.53	834.54	0.01	
81690	-	834.44	834.47	0.03	
81553	-	834.33	834.33	0.00	
			**Weighted Average Departure:**	**-0.02**	

Conclusion

Per FEMA, these tributaries are located in Floodway (Zone AE), within which no substantial rise in flood elevation is permitted. The graphical and numerical comparisons above show that the water surface elevations associated with the 100-year flood event are predicted to have a weighted average decrease of 0.02' as compared to the current condition.

References

Federal Emergency Management Agency (FEMA). https://fema.maps.arcgis.com

Federal Emergency Management Agency (FEMA). *Flood Insurance Study, Miami County, Ohio and Incorporated Areas*. October 29, 2018

Google Earth Pro V 7.3.2.5491 (64-bit). (July 23, 2018). Troy, Ohio, USA. 40° 3'53.59" N, 84°12'42.78" W, Eye alt 834 feet. [July 2018].
