# Supplementary material for: Success criteria comparison of eight implemented projects to improve the planning, design, and construction of floodplain wetlands
Source: PLOS Water. Author manuscript; Available in PMC 2026 Jun 16. (PMC13266620; doi:10.1371/journal.pwat.0000426)
Supplement: S3 File - S1 Drawings [file NIHMS2157087-supplement-S3_File_-_S1_Drawings.pdf]

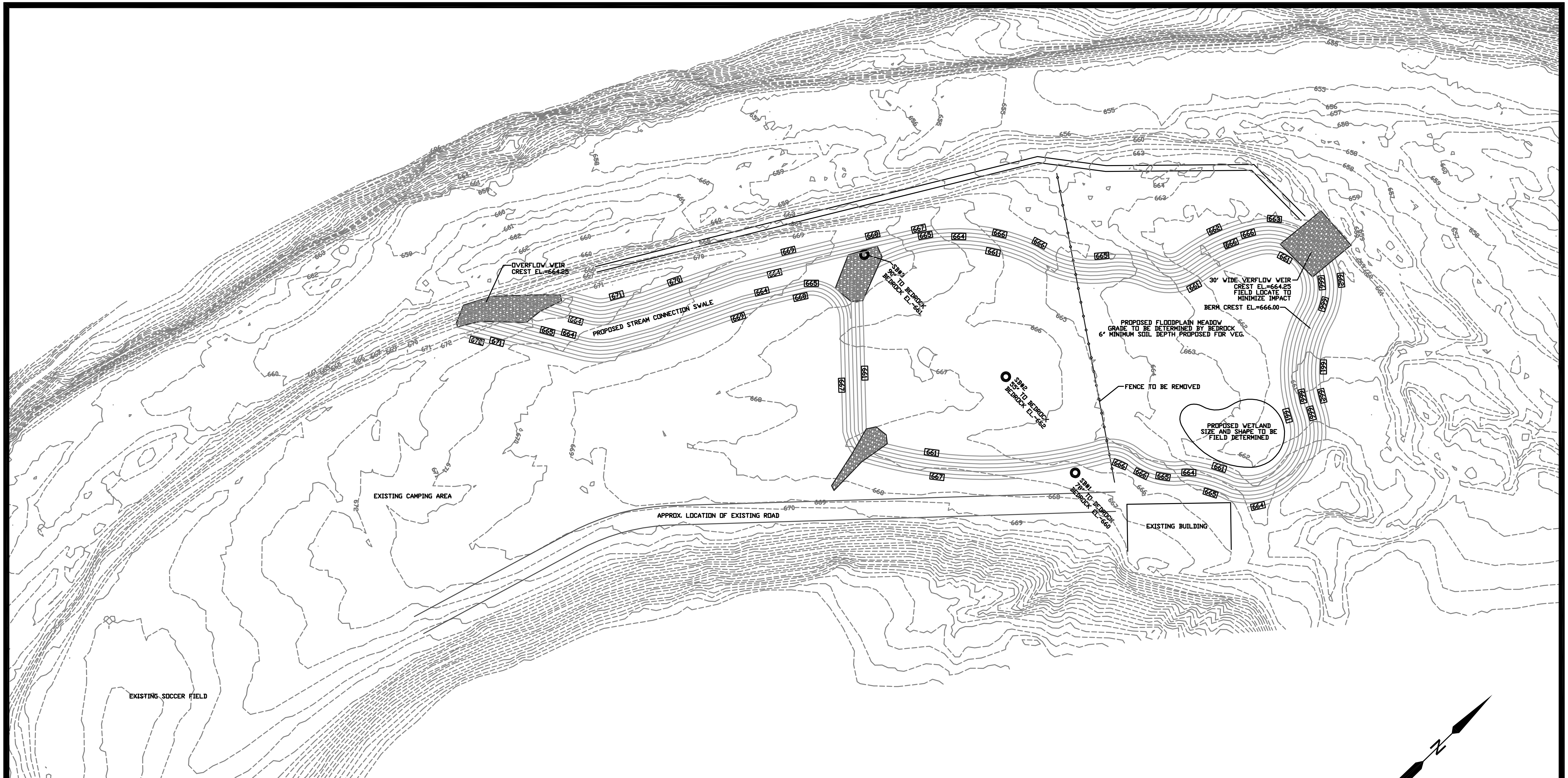

QUANTITIES

CUT – 17,200 CY
FILL – 1,600 CY
ROCK ARMORING – 600 CY

Sustainable Streams LLC
Science · Service · Solutions

PROJECT:

YMCA CAMP ERNST – BANKFULL WETLANDS ALTERNATE 1
7615 CAMP ERNST RD., BURLINGTON, KY 41005

DATE
10/8/15

DRAWN BY:
KAC

SHEET
C–1.0
