# Supplementary material for: Success criteria comparison of eight implemented projects to improve the planning, design, and construction of floodplain wetlands
Source: PLOS Water. Author manuscript; Available in PMC 2026 Jun 16. (PMC13266620; doi:10.1371/journal.pwat.0000426)
Supplement: S5 File - S2 Report [file NIHMS2157087-supplement-S5_File_-_S2_Report.pdf]

East Fork Riparian Reserve Stream and Floodplain Restoration Project Flood Risk Analysis Memorandum

Prepared for Clinton County Engineer's Office
By Sustainable Streams
December 2022

This memorandum documents the flood conveyance modeling results for the proposed stream and floodplain restoration project at the East Fork Riparian Reserve, near Lynchburg, Ohio. A portion of this project is located within the FEMA Special Flood Hazard Area (Zone A), within which no base flood elevations have been determined by FEMA. The goal of this memorandum is to provide support for the applications to permitting authorities, such as the US Army Corps of Engineers (USACE), and the Clinton County Engineer's Office, illustrating that the proposed mitigation efforts will not result in an increase to the 100-year flood elevation on adjacent or upstream properties, as compared to the existing conditions. This project aims to restore ~1,100 feet of stream and floodplain on an unnamed tributary to the East Fork Little Miami River, as well as adding ~1.4 acres of constructed wetland habitat.

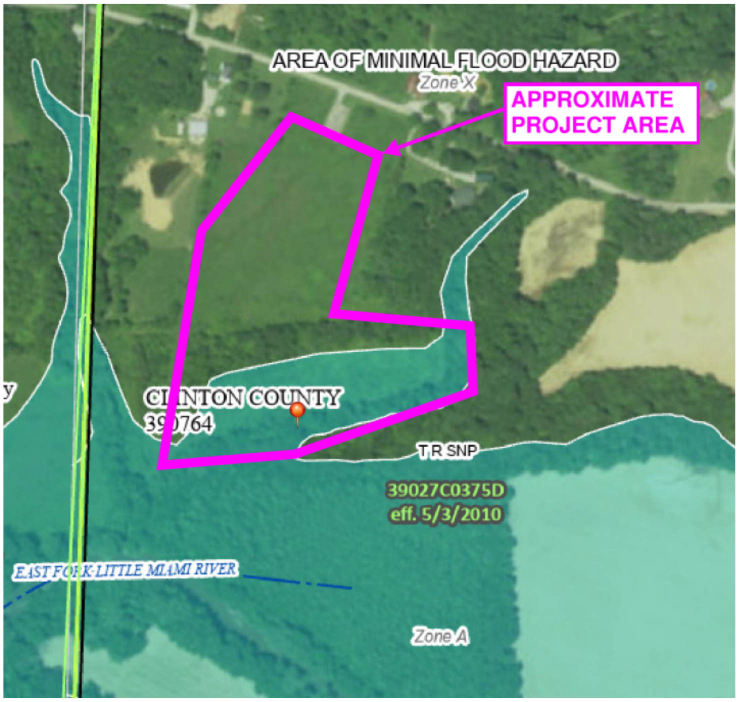

Figure 1 – FEMA Flood Insurance Map Panel 39027C0375D, dated 5/3/2010, shows a portion of the project disturbance area within Special Flood Hazard Area (Zone A) for which no base flood elevations have been determined.

Project Background

The primary goals of this project are to restore floodplain and wetland hydrology, and improve stream channel stability, water quality, aquatic habitat, and public safety. The proposed mitigation strategies include restoring a meandering pattern for the historically channelized tributary in the floodplain, regrading unstable banks to a more stable configuration, construction of habitat structures, and removal of a derelict pedestrian bridge, to be replaced with an armored low-water crossing.

Hydrologic and Hydraulic Models

Hydrology for the unnamed tributary was estimated via the use of the USGS StreamStats platform, and was used to inform channel and floodplain design, armoring, and flood analysis. For the flood analysis and channel armoring design, the 100-year discharge of 793 cfs was used.

A hydraulic model was created for the unnamed tributary to inform the local flood conveyance analysis, as well as channel and floodplain design and armoring, utilizing the U.S. Army Corps of Engineers' HEC-RAS software. As this project occurs in an off-line, backwater flood storage area adjacent to the East Fork Little Miami River, per

the FEMA flood map in **Figure 1**, a secondary, flood storage analysis was also performed, using earthwork estimates from AutoCAD Civil 3D.

Results

The local flood conveyance models indicate that the project will result in a reduction of the 100-Yr water surface elevation, with an average drop of ~1.5 feet, and no departure at the upstream and downstream ends of the project (see **Table 1** and **Figure 2**). The secondary flood storage analysis reveals that an estimated ~14,000 cubic yards of earth will be cut from within the FEMA Special Flood Hazard Area (Zone A) and placed outside it. See **Figure 3** for a representative cross section of the valley, illustrating the strategy of lowering the floodplain, which results in a net cut from the flood zone.

Table 1 – HEC-RAS modeling results for local flood conveyance at East Fork Riparian Reserve. The proposed restoration will result in an estimated average flood elevation reduction of ~1.50', with no change at the upstream or downstream ends.

East Fork Riparian Reserve Flood Modeling Profiles			
**River Station** *ft*	**Existing Q100 WSE** *ft*	**Proposed Q100 WSE** *ft*	**Q100 WSE Change** *ft*
1339.01	969.38	969.38	0.00
1297.65	968.81	968.60	-0.21
1237.56	967.97	966.76	-1.21
1227.79	968.04	966.37	-1.67
1206.07	968.04	965.67	-2.37
1065.99	967.68	965.50	-2.18
1015.98	967.55	965.31	-2.24
1005.11	967.50	964.98	-2.52
983.15	967.36	964.51	-2.85
869.03	966.87	964.55	-2.32
806.91	966.67	964.49	-2.18
795.21	966.62	964.36	-2.26
773.4	966.54	964.32	-2.22
683.82	966.23	964.30	-1.93
657.43	966.14	964.28	-1.86
644.91	966.10	964.22	-1.88
622.98	965.99	964.19	-1.80
494.41	965.56	964.21	-1.35
441.96	965.17	964.17	-1.00
430.12	965.13	964.14	-0.99
406.71	965.12	964.13	-0.99
296.82	964.73	963.61	-1.12
183.69	963.88	963.58	-0.30
169	963.76	963.51	-0.25
42.91	962.68	962.68	0.00
		**Average Q100 WSE Change (ft):**	**-1.50**

Conclusion

Per FEMA, this project is partially located within the Special Flood Hazard Area (Zone A), for which no base flood elevation has been determined. The conveyance analysis above shows that the water surface elevations associated with Q100 are predicted to decrease as compared to the current condition, with an average water surface drop of ~1.5 feet over the length of the project. Furthermore, with a net cut of ~14,000 cubic yards of soil from within the FEMA floodplain, flood levels would also be expected to decrease during backwater flood events attributable to the East Fork Little Miami River. In sum, the substantial expansion of both conveyance area and volume within the FEMA floodplain is a net improvement for flood management.

References

Federal Emergency Management Agency (FEMA) FIRM Map Number 39027C0375D (May 3, 2010). Clinton County, Ohio. https://fema.maps.arcgis.com

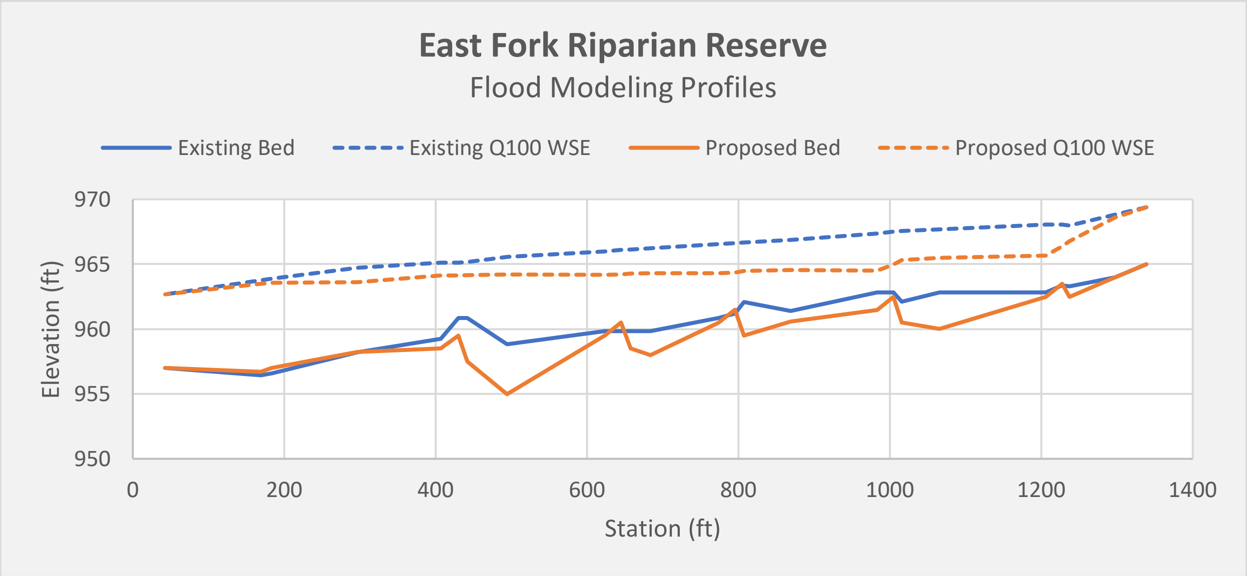

Figure 2 – HEC-RAS local flood conveyance modeling results for the unnamed tributary to the East Fork Little Miami River, located within the East Fork Riparian Reserve. The project is estimated to result in an average flood elevation reduction of ~1.5 ft with no change at the upstream or downstream ends.

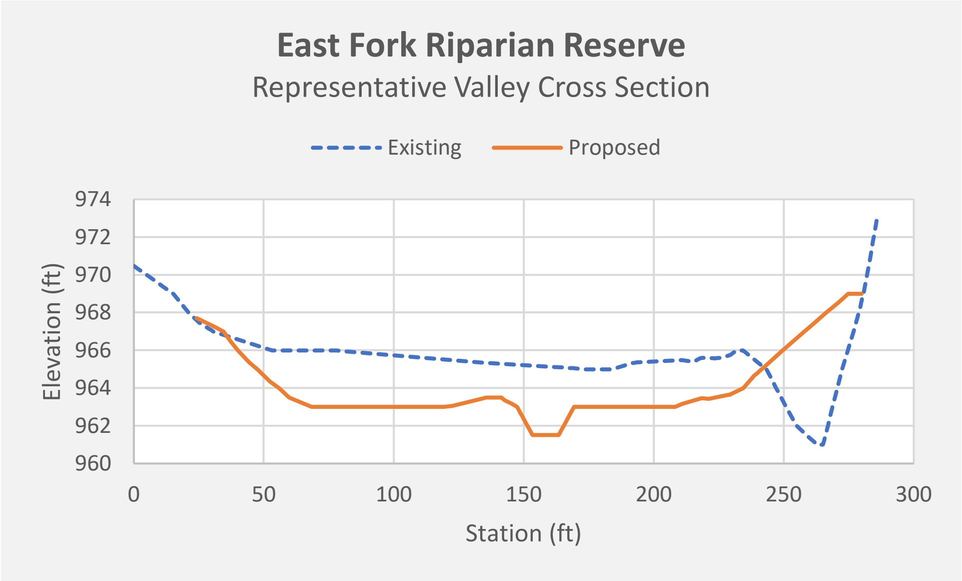

Figure 3 – Valley cross section at profile station 7+95, looking upstream, illustrating the net cut from within the floodplain. The excess earth will be hauled outside the FEMA Special Flood Hazard Area (Zone A) and used for trail and other improvements within the park property.
